# Supplementary material for: BLM has Contrary Effects on Repeat-Mediated Deletions, based on the Distance of DNA DSBs to a Repeat and Repeat Divergence
Source: Cell Rep. Author manuscript; Available in PMC 2020 Mar 21. (PMC7085117; doi:10.1016/j.celrep.2020.01.001)
Supplement: 1 [file NIHMS1568954-supplement-1.pdf]

Cell Reports, Volume *30*

Supplemental Information

BLM has Contrary Effects on Repeat-Mediated Deletions, based on the Distance of DNA DSBs to a Repeat and Repeat Divergence

Carlos Mendez-Dorantes, L. Jillianne Tsai, Eva Jahanshir, Felicia Wednesday Lopezcolorado, and Jeremy M. Stark

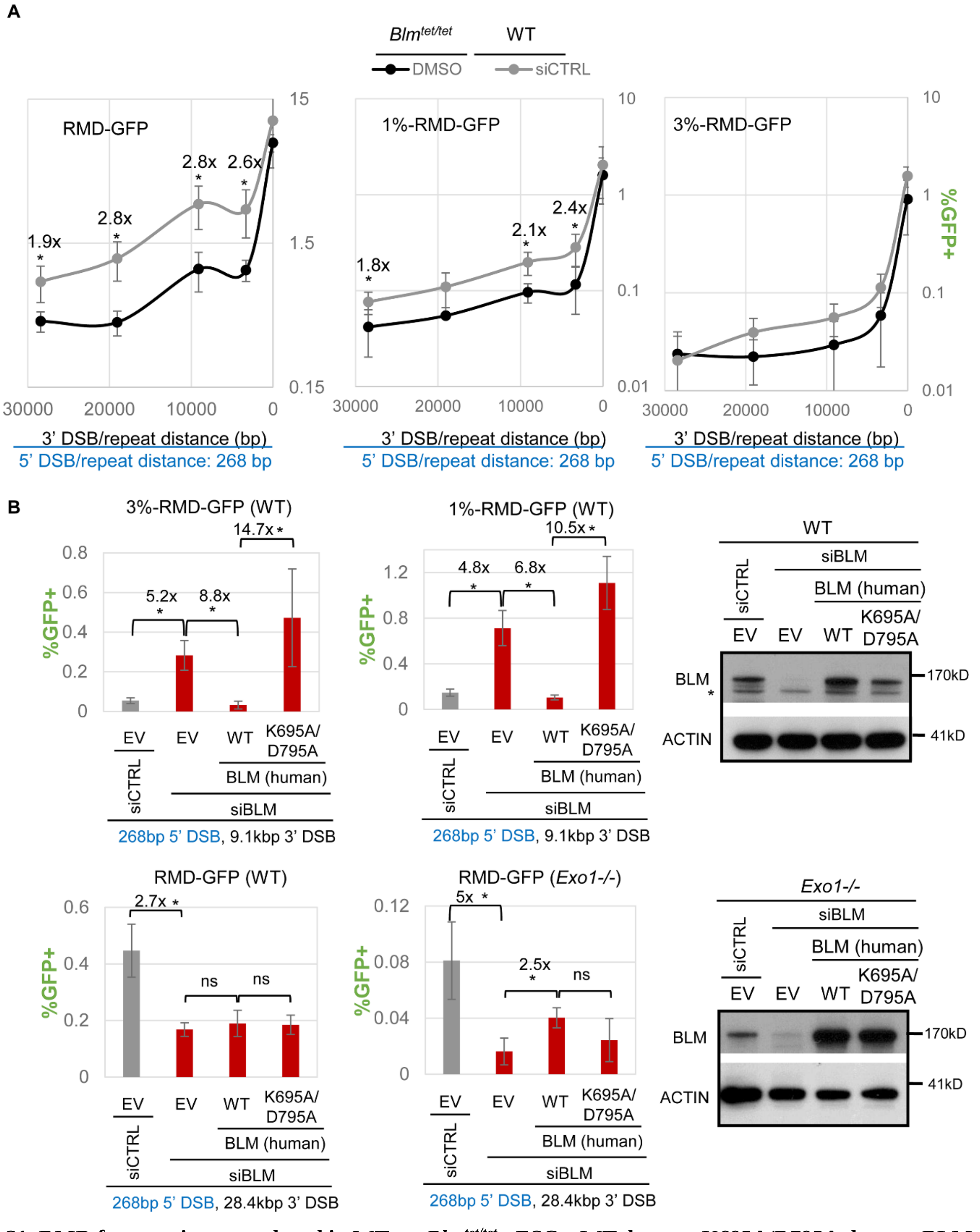

Figure S1. RMD frequencies are reduced in WT vs. *Blm*$^{tet/tet}$mESCs; WT, but not K695A/D795A, human BLM suppresses RMDs between 1% or 3% divergent repeats at the 9.1 kbp 3'-DSB/repeat distance, Related to Figure 1. (A) Comparisons of WT (siCTRL treated) vs. *Blm*$^{tet/tet}$ (DMSO treated) mESCs for each RMD reporter (identical, 1%, and 3%). Frequencies are the same as in Figure 1. N=6. *$P \leq 0.0266$ for WT siCTRL treated vs. *Blm*$^{tet/tet}$ DMSO treated, except at 28.4 kbp between 1% divergent repeats where $P = 0.0487$. (B) Frequencies of specific RMDs for cells treated with siCTRL or siBLM and transfected with various expression vectors (human BLM WT, the K695A/D795A mutant, and EV). WT N=6. *Exo1-/-* N=5. *$P \leq 0.0041$. Immunoblotting analysis of BLM and ACTIN of cells treated with siRNA, and transfected with these vectors. *nonspecific band.

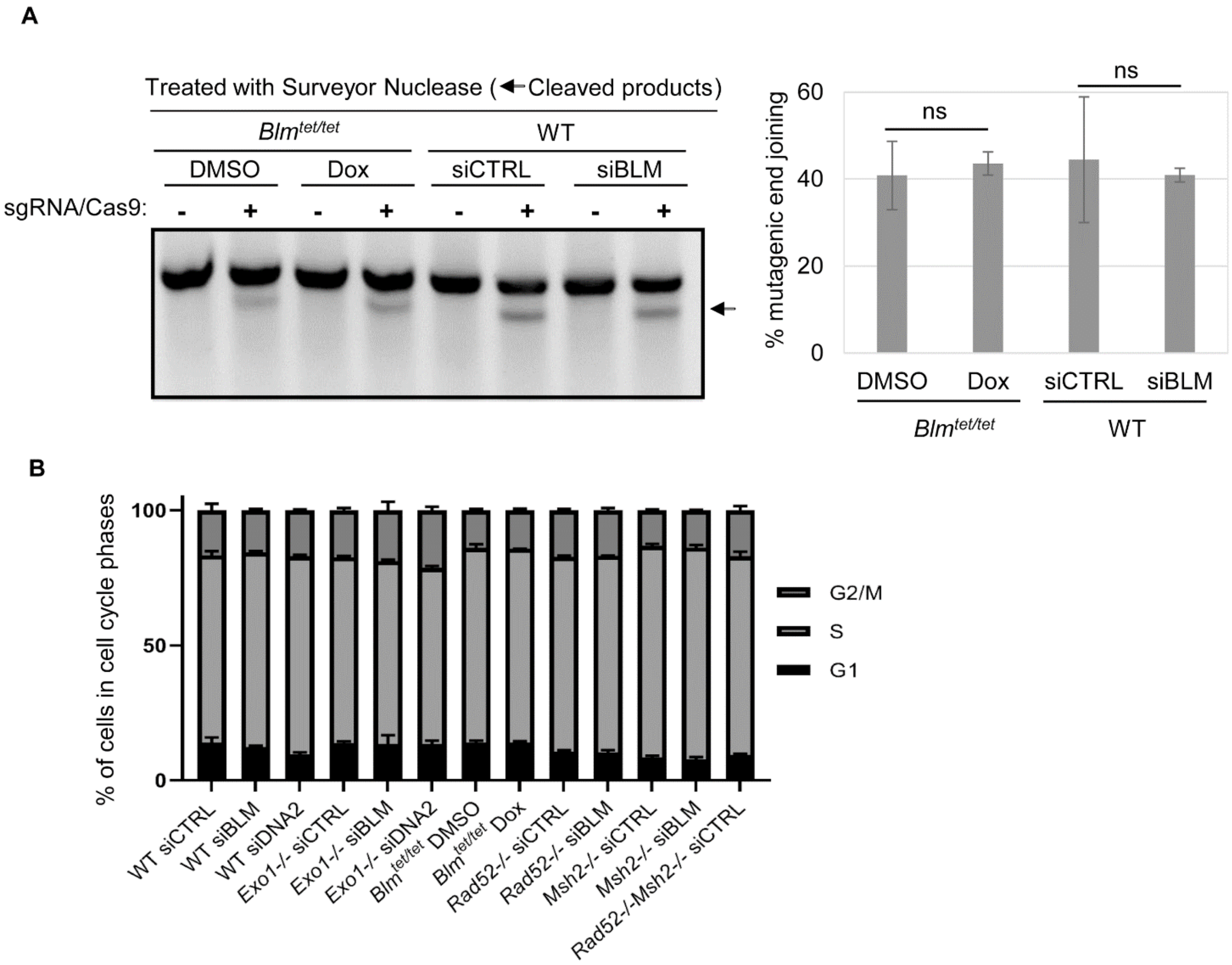

Figure S2. BLM depletion does not obviously affect Cas9-mediated mutagenic end joining; cell cycle profiles are not obviously affected by the genetic depletions/disruptions of various factors, Related to Figure 1. (A) Representative Surveyor nuclease assays from cells transfected with the 19 kbp sgRNA/Cas9 expression plasmid (sgRNA/Cas9). Arrow indicates the Surveyor cleaved product. Shown is the frequency of mutagenic end joining from replicates of this assay, normalized to transfection efficiency. N=3. ns= not significant. (B) Cell cycle profiles (G1, S and G2/M) of the mESC lines under various genetic depletions/disruptions, using BrdU labeling and propidium iodide staining. N=3.

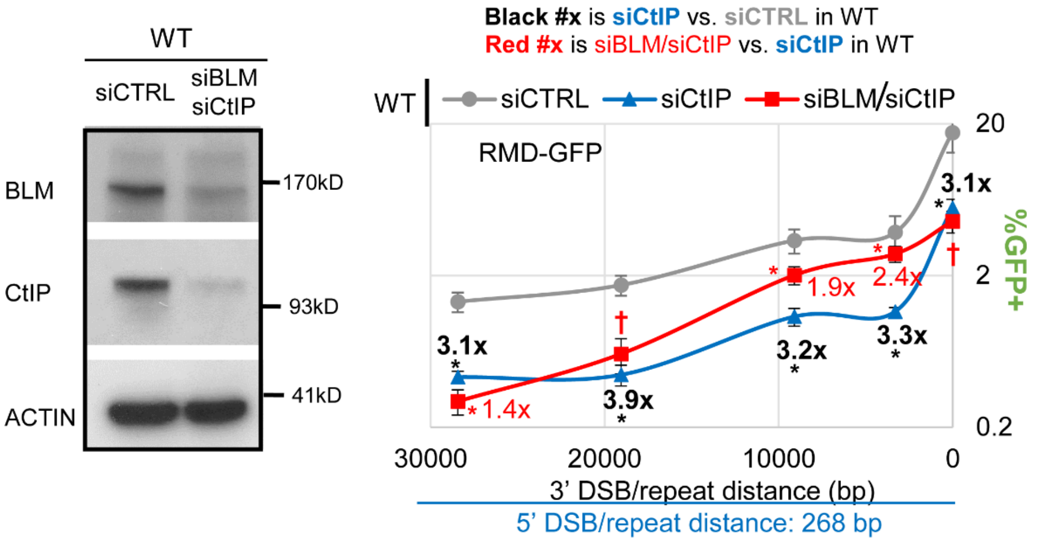

Figure S3. Influence of CtIP depletion alone, and in combination with BLM depletion, on RMDs with identical repeats, Related to Figure 2. RMD frequencies for cells treated with four siRNAs targeting CtIP (siCtIP) alone, or in combination with siBLM (siBLM/siCtIP), along with non-targeting siCTRL. N=6. **P*≤0.00275, or †*P*-unadjusted≤0.041 for the indicated comparisons. Immunoblotting analysis of CtIP, BLM, and ACTIN from cells treated with these siRNAs.

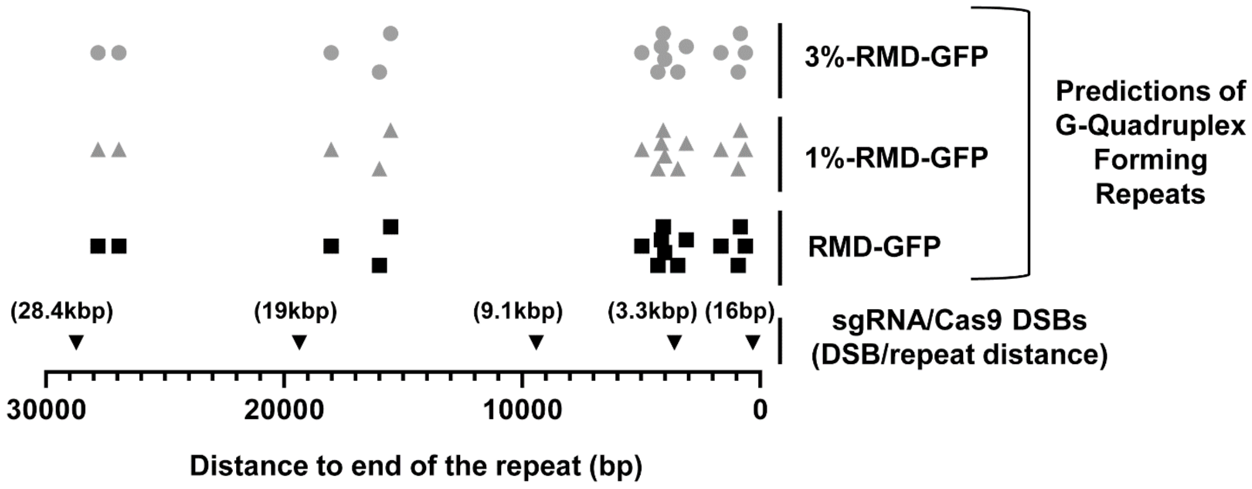

Figure S4. Predictions of G-quadruplex forming repeats in the RMD-GFP reporter assay sequences, Related to Figure 6. Shown are predictions of G-quadruplex forming repeats in the edge of the 28.4 kbp DSB to the end of each repeat sequence (identical, 1% or 3%) in the RMD-GFP reporters, based on the sequence of chromosome 17 from Genome Reference Consortium Mouse Build 38 (GCA_000001635.2), and using the non-B DNA Motif Search Tool (Cer et al., 2013). Also shown are the locations of the sgRNA/Cas9-mediated DSBs.

Table S1. List of oligonucleotide sequences used in this study, Related to STAR Methods.

**Reagent**	**Sequence**
DRGFP sgRNA target	5' GGATAACAGGGTAATACCTA
5’268 sgRNA target	5’ GCCATAGGCGTGGGACCTCGT
16 bp sgRNA target	5’ GATCAGAAGCTTAGGGATAAC
3.3 kbp sgRNA target	5’GCGCCGCACCGACACCCTGG
9.1 kbp sgRNA target	5’ GATGTTTGGATTGTATGGCCA
19 kbp sgRNA target	5’ GTCCTGCCATAGCCTTGATG
28.4 kbp sgRNA target	5’ GCGAAGCTAACGACACTAACG
Msh2 sgRNA 1	5' GTTGGAAGGCGCGGCCGAGG
Msh2 sgRNA 2	5’ GACGGTGCGCCTCTTCGACCG
Msh2 screening P1	5’ CGGTGCAGCCTAAGGAGAC
Msh2 screening P2	5’ CGGCCCCATGTACTTGAT
Pim1 sgRNA 1	5’ GCTCGGGCGTCATTAGACTTC
Pim1 screening P1	5’ CTAACCCTCGCCCTCACTCT
Pim1 screening P2	5’ CTGGCTCACCATCAAAGTCC
DNA2 RT-PCR P1	5’ GCGTTCTGCTGACCAGCTAC
DNA2 RT-PCR P2	5’ CTGGATGTCTGGATGGACCT
ACTIN RT-PCR P1	5’ GGCTGTATTCCCCTCCATCG
ACTIN RT-PCR P2	5’ CCAGTTGGTAACAATGCCATGT
Surveyor P1	5’CAGCGAACAGGTCGGTTAAT
Surveyor P2	5’ATCACTTGCTCAAGCACACG
non-targeting siCTRL D-001810-01	5' UGGUUUACAUGUCGACUAA
siBLM D-061987-01 target	5' GACACAAUCUGAAGUACUA
siBLM D-061987-02 target	5 'CUAAAUCUAUGGAGGGUUA
siBLM D-061987-03 target	5' CCUAUGAUAUCGAUAACUU
siBLM D-061987-04 target	5' ACACCUGCGUUAAGUGAUA
siDNA2 D-062864-01 target	5' CGACAUAAGUACUCCACUA
siDNA2 D-062864-02 target	5' CGAGGUCCAUUGCGUCUUU
siDNA2 D-062864-03 target	5' GCACUCCGCCGUUGACAAU
siDNA2 D-062864-04 target	5' AAUAGACGAUGACUUUGGA
siCtIP D-055713-14 target	5' CCUAGACACUGGCGUGAAA
siCtIP D-055713-15 target	5' GCAUUAACCGGCUACGAAA
siCtIP D-055713-16 target	5' AUAUUGAGGUAGUUCGGAA
siCtIP D-055713-17 target	5' AGAUAUGUUUGAUCGGACA
siBRCA2 J-042993-05 target	5' GAACAUAGUUUGGUCAUUA
siBRCA2 J-042993-06 target	5' CUAAGCAGCUCUAUAUAUA
siBRCA2 J-042993-07 target	5' GGAGGUAAAUGAAAUAUGU
siBRCA2 J-042993-08 target	5' GAUCCAGUCUUGAACCAUA
