# Supplementary material for: BLM has Contrary Effects on Repeat-Mediated Deletions, based on the Distance of DNA DSBs to a Repeat and Repeat Divergence
Source: Cell Rep. Author manuscript; Available in PMC 2020 Mar 21. (PMC7085117; doi:10.1016/j.celrep.2020.01.001)
Supplement: 2 [file NIHMS1568954-supplement-2.pdf]

Cell Reports

Article

BLM has Contrary Effects on Repeat-Mediated Deletions, based on the Distance of DNA DSBs to a Repeat and Repeat Divergence

Graphical Abstract

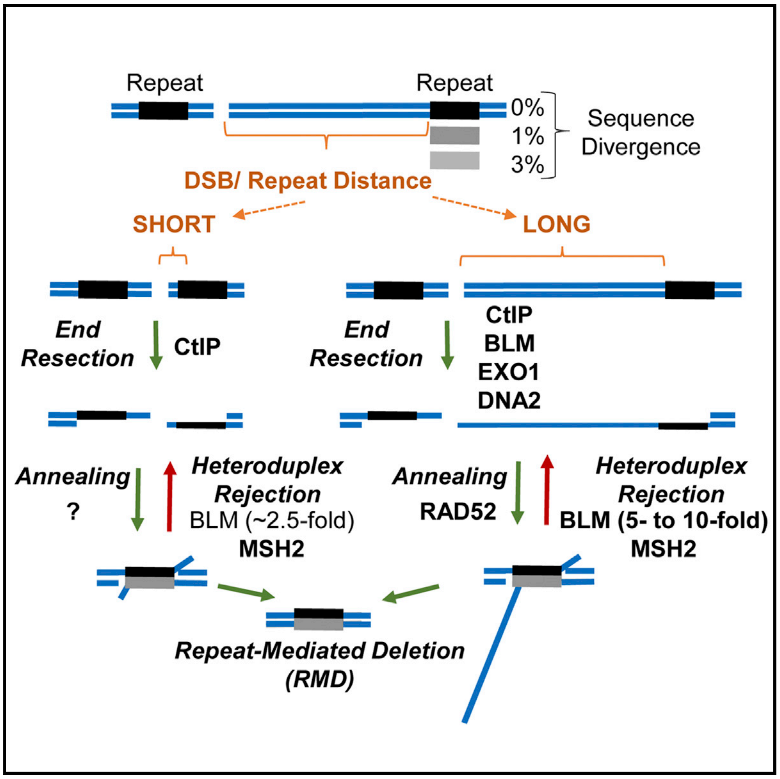

Highlights

- DNA break/repeat distance and repeat divergence specify RMD mechanisms
- BLM, EXO1, and DNA2 mediate RMDs with remarkably long DNA break/repeat distances
- BLM heteroduplex rejection is optimal with a long non-homologous tail
- BLM heteroduplex rejection is independent of MSH2 and RAD52

Authors

Carlos Mendez-Dorantes, L. Jillianne Tsai, Eva Jahanshir, Felicia Wednesday Lopezcolorado, Jeremy M. Stark

Correspondence

jstark@coh.org

In Brief

Mendez-Dorantes et al. identify the BLM helicase as a key regulator of repeat-mediated deletions (RMDs). BLM, EXO1, and DNA2 mediate RMDs with remarkably long DNA break/repeat distances. BLM suppresses RMDs with sequence divergence that is optimal with a long non-homologous tail and is independent of MSH2 and RAD52.

Mendez-Dorantes et al., 2020, Cell Reports *30*, 1342–1357
February 4, 2020 © 2020 The Author(s).
https://doi.org/10.1016/j.celrep.2020.01.001

OPEN ACCESS
CellPress

Cell Reports
Article

BLM has Contrary Effects on Repeat-Mediated Deletions, based on the Distance of DNA DSBs to a Repeat and Repeat Divergence

Carlos Mendez-Dorantes,[1,2] L. Jillianne Tsai,[1,2] Eva Jahanshir,[1] Felicia Wednesday Lopezcolorado,[1] and Jeremy M. Stark[1,2,3,*]
[1]Department of Cancer Genetics and Epigenetics, Beckman Research Institute of the City of Hope, Duarte, CA 91010, USA
[2]Irell and Manella Graduate School of Biological Sciences, Beckman Research Institute of the City of Hope, Duarte, CA 91010, USA
[3]Lead Contact
*Correspondence: jstark@coh.org
https://doi.org/10.1016/j.celrep.2020.01.001

SUMMARY

Repeat-mediated deletions (RMDs) often involve repetitive elements (e.g., short interspersed elements) with sequence divergence that is separated by several kilobase pairs (kbps). We have examined RMDs induced by DNA double-strand breaks (DSBs) under varying conditions of repeat sequence divergence (identical versus 1% and 3% divergent) and DSB/repeat distance (16 bp–28.4 kbp). We find that the BLM helicase promotes RMDs with long DSB/repeat distances (e.g., 28.4 kbp), which is consistent with a role in extensive DSB end resection, because the resection nucleases EXO1 and DNA2 affect RMDs similarly to BLM. In contrast, BLM suppresses RMDs with sequence divergence and intermediate (e.g., 3.3 kbp) DSB/repeat distances, which supports a role in heteroduplex rejection. The role of BLM in heteroduplex rejection is not epistatic with MSH2 and is independent of the annealing factor RAD52. Accordingly, the role of BLM on RMDs is substantially affected by DSB/repeat distance and repeat sequence divergence.

INTRODUCTION

Mammalian genomes contain a high density of repetitive DNA elements, such as long interspersed elements and short interspersed elements (Ade et al., 2013; de Koning et al., 2011). Indeed, the human genome includes approximately one million copies of *Alu*-like short interspersed elements (Batzer and Deininger, 2002; Burns, 2017; de Koning et al., 2011; Deininger, 2011; Treangen and Salzberg, 2011). Such repetitive elements have the potential to recombine, thereby causing chromosomal rearrangements. In particular, two repetitive elements on the same chromatid can recombine to cause a deletion rearrangement, which we refer to as a repeat-mediated deletion (RMD). RMDs have been found to cause several rearrangements associated with human disease (Deininger and Batzer, 1999). For example, RMDs between *Alu* elements have been found to disrupt tumor suppressor genes, such as *BRCA1* and *MSH2* (Kolomietz et al., 2002; Pavlicek et al., 2004; Pérez-Cabornero et al., 2011).

RMD events in humans can span a wide array of distances between the repeats, as well as varying degrees of homology between the repeats (i.e., sequence divergence). A survey of >200 rearrangements involving two *Alu* elements, used to develop a predictive computational model for such rearrangements, showed that *Alu*-mediated deletions can be as short as <1 kilobase pair (kbp) to over 4 megabase pairs (Mbp), although the vast majority were <57 kbp (Song et al., 2018). Furthermore, this survey of *Alu*-mediated rearrangements uncovered a bias for events involving evolutionarily younger *Alu* elements (Song et al., 2018), which generally have low sequence divergence (Batzer and Deininger, 2002). Accordingly, examining how repeat distance and sequence divergence affect RMD mechanisms will provide insight into the etiology of these rearrangements. Similarly, the distance between the initiating DNA lesion(s) and each of the repeats likely affects the mechanism of RMDs.

One model for RMD formation is repair of a DNA double-strand break (DSB) that uses annealing of two flanking repeats to bridge the DSB, resulting in the deletion of one of the repeats and the intervening sequence. This model for RMD formation is referred to as single-strand annealing (SSA) (Bhargava et al., 2016; Morales et al., 2018). Based on this model, a key step of RMD formation that is affected by DSB/repeat distance is end resection, which refers to the processing of DSBs into 3′ single-stranded DNA (ssDNA) that reveals the repeats used for repair (Symington and Gautier, 2011). As the distance between the DSB and each repeat increases, so does the length of end resection that is required for each repeat to be revealed in ssDNA for the annealing step. Consistent with this model, factors important for end resection promote RMDs, including CtIP and its ortholog *SAE2* in the yeast *Saccharomyces cerevisiae* (*S. cerevisiae*) (Bennardo et al., 2008; Mendez-Dorantes et al., 2018; Mimitou and Symington, 2008; Sartori et al., 2007; Zhu et al., 2008). Also in *S. cerevisiae*, the nucleases *EXO1* and *DNA2*, along with the *SGS1* RecQ-helicase, are important for extensive end resection and RMD events (Mimitou and Symington, 2008; Zhu et al., 2008).

Also based on the SSA model, after end resection, the repeats are synapsed to form an annealing intermediate. When divergent

 © 2020 The Author(s).
This is an open access article under the CC BY license (http://creativecommons.org/licenses/by/4.0/).

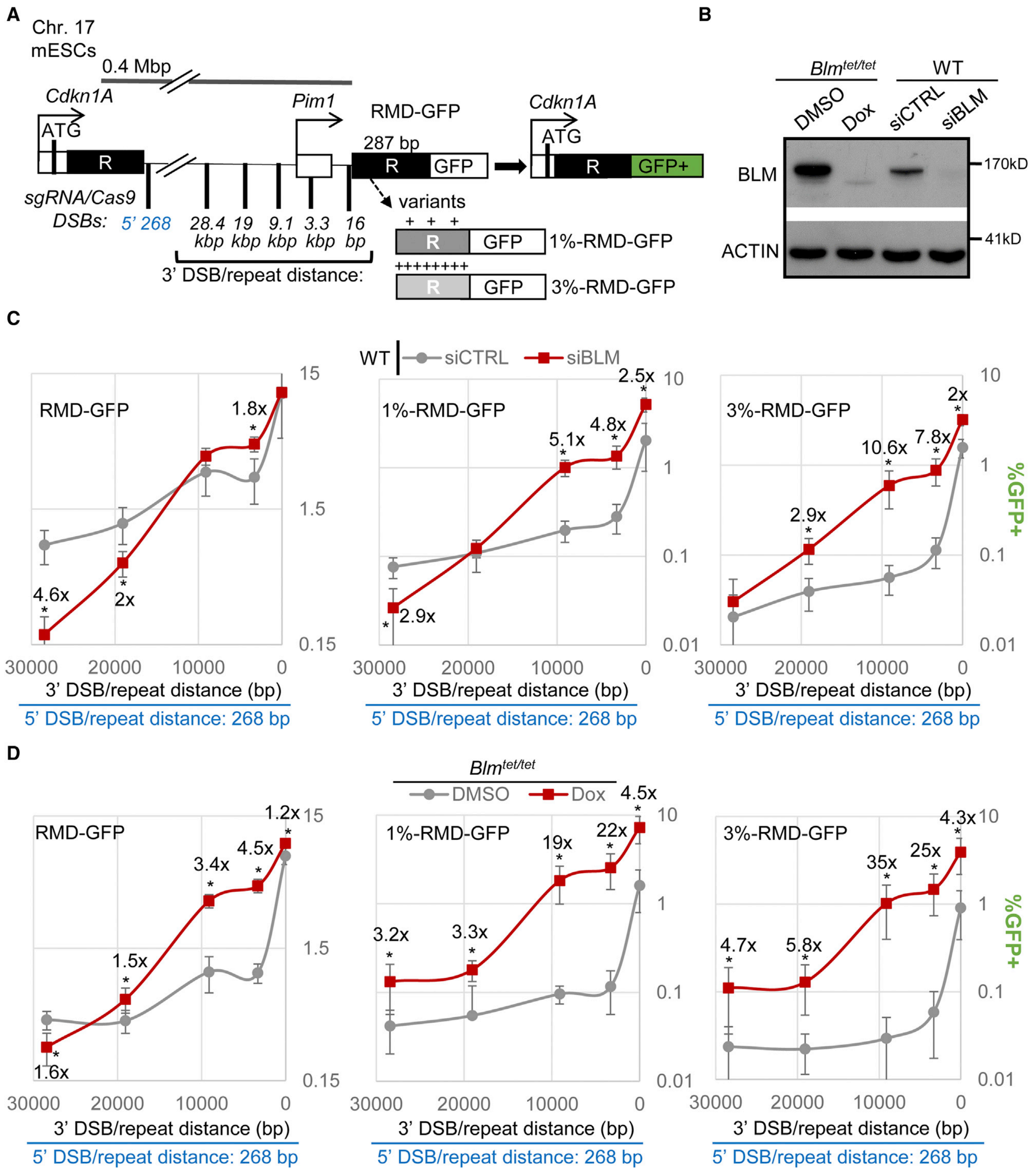

Figure 1. BLM Promotes RMDs Induced by Chromosomal DSBs Far from a Homologous Repeat but Markedly Suppresses RMDs with Intermediate DSB/Repeat Distances, Particularly with Divergent Repeats

(A) A schematic of the RMD-GFP reporter with two repeats on chromosome 17 of mouse embryonic stem cells (mESCs). An RMD causes GFP expression. 1%-RMD-GFP and 3%-RMD-GFP have 1% and 3% sequence divergence between the repeats, respectively. RMDs are induced with two sgRNA/Cas9-mediated DSBs: one DSB is 268 bp downstream of the 5′ repeat, and the second DSB is various distances upstream of the 3′ repeat.

(legend continued on next page)

repeats are annealed together, the double-stranded DNA (dsDNA) contains mismatched bases and hence forms a heteroduplex. This intermediate is prone to reversal by heteroduplex rejection, which is mediated by proteins in the mismatch repair pathway (Alani et al., 1994; Goldfarb and Alani, 2005; Sugawara et al., 2004; Waldman and Liskay, 1988). For example, MSH2 is important to suppress RMDs, and other homologous recombination events, between divergent sequences (Elliott and Jasin, 2001; Goldfarb and Alani, 2005; Mendez-Dorantes et al., 2018; Sugawara et al., 2004). Another factor important for heteroduplex rejection in *S. cerevisiae* is *SGS1* (Goldfarb and Alani, 2005; Spell and Jinks-Robertson, 2004; Sugawara et al., 2004). However, as mentioned above, *SGS1* also is important for end resection and as such appears to have contrary roles in RMD formation in yeast.

The mammalian ortholog of *SGS1* that influences these steps of RMDs (i.e., end resection and/or heteroduplex rejection) has been unclear, because there are five mammalian RecQ-helicases (Croteau et al., 2014). A possible candidate is the BLM helicase, which is found mutated in the inherited disease Bloom's syndrome (Ellis et al., 1995). BLM has long been linked to suppression of homologous recombination, because BLM-deficient cell lines show a high frequency of sister chromatid exchanges (Chaganti et al., 1974). The BLM protein can unwind a variety of DNA structures, including displacement loop recombination intermediates (Bachrati et al., 2006). This unwinding activity is likely central to BLM-mediated suppression of sister chromatid exchanges and furthermore has recently been implicated in dissolution of recombination intermediates during alternative lengthening of telomeres (Lu et al., 2019; Panier et al., 2019; Silva et al., 2019). However, BLM was shown to have no obvious effect on DSB-induced homologous recombination in mouse and human cells, including between ∼1.5% divergent sequences (Larocque and Jasin, 2010). Although, BLM was shown to inhibit DSB-induced homologous recombination events in human cells involving repeats with 19% sequence divergence (Wang et al., 2016), indicating a possible role for BLM on heteroduplex rejection with very high sequence divergence. However, the parameters that determine the role of BLM on heteroduplex rejection are poorly defined. Regarding the end resection step of RMDs, although BLM is substantially active in biochemical assays for this process (Daley et al., 2017; Nimonkar et al., 2011; Soniat et al., 2019), the relative importance of this factor in cells to promote end resection during chromosomal DSB repair is unclear.

We posited that the influence of BLM on RMD formation may be affected by DSB/repeat distance and repeat divergence. Based on the SSA model, the DSB/repeat distance defines the length of end resection that is required for an RMD. Furthermore, this DSB/repeat distance affects the length of the non-homologous flanking sequence in the annealing intermediate, which we posited might influence heteroduplex rejection. Thus, we have examined how these various features of RMDs affect the relative role of BLM and other factors (e.g., EXO1, DNA2, MSH2, and the DNA annealing factor RAD52) on these repair events.

RESULTS

BLM Promotes RMDs Induced by Chromosomal DSBs Far from a Homologous Repeat but Markedly Suppresses RMDs with Intermediate DSB/Repeat Distances, Particularly with Divergent Repeats

We have investigated the influence of the BLM helicase on RMD formation. For this, we used a previously described reporter system (RMD-GFP; Figure 1A), in which a 287-bp fragment of the *Cdkn1a* gene is fused to GFP and integrated into the *Pim1* locus on chromosome 17 in mouse embryonic stem cells (mESCs) (Mendez-Dorantes et al., 2018). Introducing this 287-bp fragment of the *Cdkn1a* gene at the *Pim1* locus creates two tandem repeats, (shown as R in Figure 1A), which are similar in size to human *Alu* elements (Batzer and Deininger, 2002). An RMD event between these two repeats generates a *Cdkn1a-GFP* fusion gene, causing GFP expression that can be detected with flow cytometry. The *Cdkn1a* locus is 0.4 Mbp upstream of the *Pim1* locus, such that we generate two Cas9 DSBs to induce RMDs: the first DSB is 268 bp downstream of the 5′ repeat in the *Cdkn1a* locus and the second DSB is various distances upstream of the 3′ repeat in the *Pim1* locus (3′-DSB/repeat distances of 16 bp, 3.3 kbp, 9.1 kbp, 19 kbp, and 28.4 kbp). We created two variants of this assay, in which the 3′ repeat has three and eight point mutations, which causes 1% and 3% sequence divergence, respectively. In a prior study from our laboratory, we validated the single-guide RNAs (sgRNAs) targeting Cas9-induced DSBs to the various positions, as well as the structure of the RMD events from sorted GFP+ cells (Mendez-Dorantes et al., 2018).

To examine the influence of BLM on these RMD events, we treated cells with a pool of four small interfering RNAs (siRNAs) targeting BLM (siBLM), which we confirmed depletes BLM via immunoblotting (Figure 1B). Control cells are treated with non-targeting siRNA (siCTRL). We first tested RMDs with identical repeats and found that siBLM had no effect on these events at the 3′-DSB/repeat distances of 16 bp and 9.1 kbp but caused an increase for the 3.3-kbp distance (Figure 1C; 1.8-fold). In contrast, we found that siBLM caused a reduction in RMDs for the 19-kbp and 28.4-kbp 3′-DSB/repeat distances (2-fold and 4.6-fold, respectively). We then examined RMDs with 1% and 3% sequence divergence. For the 1% divergent RMDs, siBLM caused a marked increase for these events for 16-bp, 3.3-kbp, and 9.1-kbp 3′-DSB/repeat distances (2.5-, 4.8-, and 5.1-fold, respectively). In contrast, siBLM had no effect for the 19-kbp distance and, as with the identical repeats, caused a reduction for the 28.4-kbp distance (2.9-fold). For the 3% divergent RMDs, siBLM caused a substantial increase in these events for the 16-bp, 3.3-kbp, and 9.1-kbp 3′-DSB/repeat

(B) Immunoblotting analysis of BLM and ACTIN in WT mESCs treated with siBLM, $Blm^{tet/tet}$ mESCs with Dox treatment, and control treatments with siCTRL or DMSO.

(C) Effects of siBLM treatment on RMDs. n = 6. *$p \leq 0.0119$ for siBLM versus siCTRL. Shown are fold effects of significant differences, using the mean.

(D) RMDs in $Blm^{tet/tet}$ mESCs treated with Dox (BLM depleted) versus DMSO (BLM overexpression versus WT). n = 6. *$p \leq 0.0229$ for Dox versus DMSO. Fold effects as in (C) are shown.

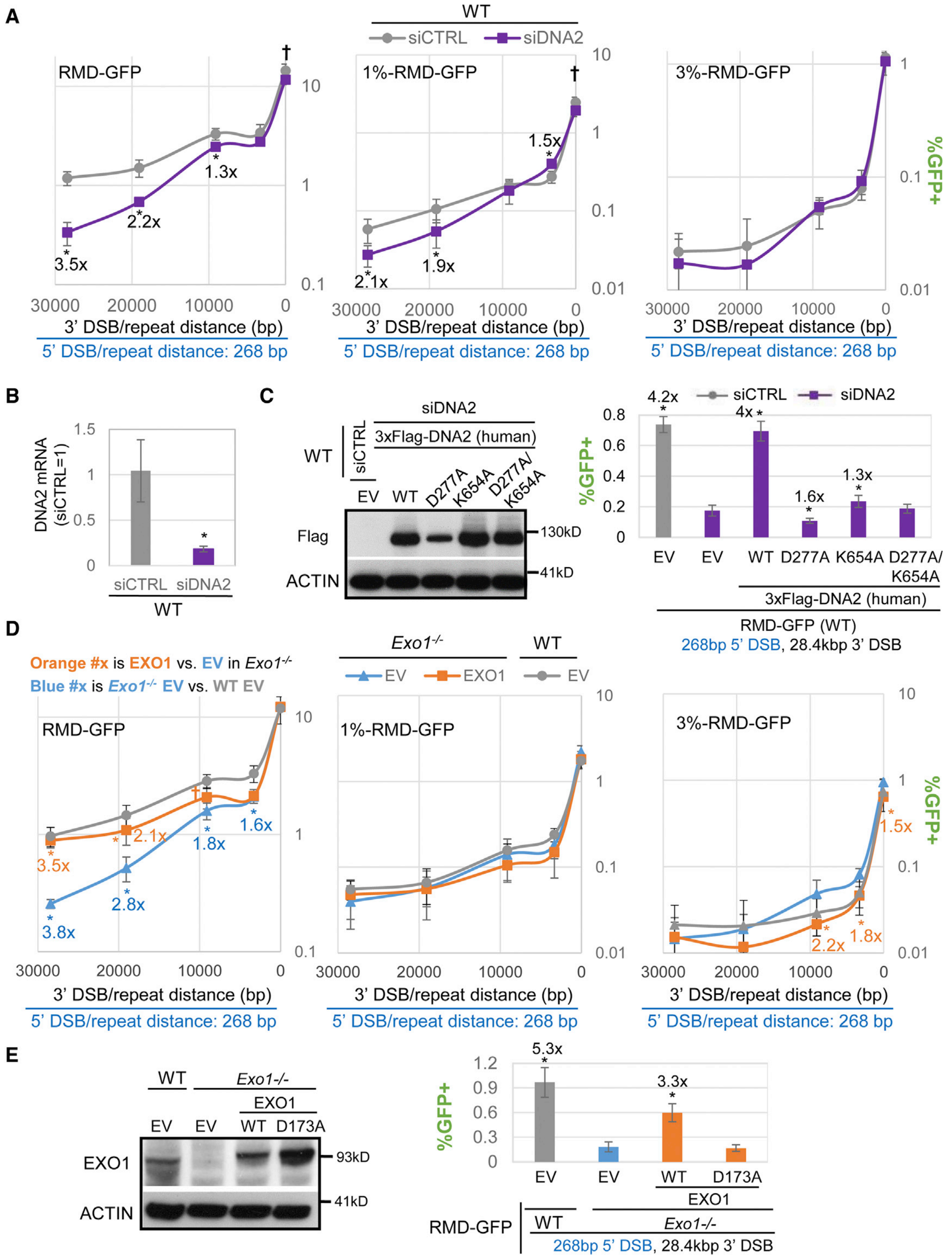

Figure 2. The DSB End Resection Factors EXO1 and DNA2 Are Particularly Important for RMDs with Long 3′-DSB/Repeat Distances, Similar to BLM

(A) Effects of siDNA2 treatment on RMDs. n = 6. *p ≤ 0.0374 and †p-unadjusted ≤ 0.0355 for siCTRL versus siDNA2. Shown are fold effects of significant differences, using the mean.

(B) RT-PCR analysis of DNA2 mRNA in WT mESCs treated with siDNA2 or siCTRL. n = 3 PCR. *p = 0.0124.

(C) Effects of 3×FLAG-DNA2 expression vectors on the 28.4-kbp RMD with identical repeats in cells treated with siDNA2. n = 6. *p ≥ 0.041 versus siDNA2 EV. Immunoblotting analysis of FLAG and ACTIN using these vectors. Fold effects as in (A) are shown.

(legend continued on next page)

distances (2-, 7.8-, and 10.6-fold, respectively) but, in this case, also caused an increase at 19 kbp (2.9-fold) and had no effect at 28.4 kbp (Figure 1C). Notably, for both 1% and 3% divergent RMDs, siBLM caused a greater increase for the intermediate DSB/repeat distances (3.3 kbp and 9.1 kbp) than the short distance (16 bp).

We also examined BLM using a different method: an mESC line that allows for repression of endogenous BLM expression using doxycycline (Dox) treatment, due to replacement of the *Blm* promoter on both alleles with a Dox-responsive cassette ($Blm^{tet/tet}$ mESCs; Yusa et al., 2004). We confirmed that Dox treatment in $Blm^{tet/tet}$ mESCs causes depletion in BLM protein, compared to vehicle (DMSO; Figure 1B). We also found that, in the control conditions (DMSO), BLM levels are substantially higher than wild-type (WT) mESCs (Figure 1B), which is likely due to the Dox-responsive cassette that uses the tetracycline transactivator (Bond et al., 2000). Accordingly, this cell line facilitates a comparison of overexpression of BLM versus loss of BLM (control versus Dox-treated, respectively). With this $Blm^{tet/tet}$ mESC line, the RMD reporters were integrated and cells were pre-treated with DMSO or Dox prior to transfection with the sgRNA/Cas9 plasmids and then treated with DMSO or Dox until analysis.

We first tested the $Blm^{tet/tet}$ cell line with the RMD reporter with identical repeats and found that Dox treatment caused a marked increase in RMD frequency at the 3′-DSB/repeat distances of 3.3 kbp and 9.1 kbp (Figure 1D; 4.5-fold and 3.4-fold, respectively). Furthermore, Dox treatment caused a modest increase in RMD frequencies at the 3′-DSB/repeat distances of 16 bp and 19 kbp (Figure 1D; 1.2- and 1.5-fold, respectively). In contrast, we found that Dox treatment caused a decrease in RMD frequency at the 28.4-kbp 3′-DSB/repeat distance (1.6-fold). With both 1% and 3% sequence divergence, we found that Dox treatment caused a significant increase in RMDs at all 3′-DSB/repeat distances; however, the fold effects were affected by the distance. Specifically, Dox treatment caused a dramatic increase in RMDs with 1% and 3% divergence for the 3.3-kbp and 9.1-kbp distances, but the effects were lower for the 16-bp, 19-kbp, and 28.4-kbp distances (Figure 1D).

We then compared the findings between these two approaches for manipulating BLM levels. As mentioned above, the control condition for the $Blm^{tet/tet}$ mESCs reflects overexpression of BLM compared to WT. Consistent with elevated levels of BLM, $Blm^{tet/tet}$ mESCs show lower RMD frequencies for several events compared to WT (Figure S1A). In comparing fold effects between the two approaches, we found that Dox treatment in the $Blm^{tet/tet}$ mESCs caused a more dramatic increase in RMDs compared to siBLM (Figures 1C and 1D). However, the overall pattern of effects of 3-DSB/repeat distance and repeat divergence were similar (Figures 1C and 1D). With both methods, BLM has the most profound effect on inhibiting RMDs at intermediate 3′-DSB/repeat distances (i.e., 3.3 kbp and/or 9.1 kbp), which was substantially magnified with sequence divergence. In contrast, this inhibitory effect of BLM was diminished for the short 3′-DSB/repeat distance (i.e., 16 bp). Finally, for the long 3′-DSB/repeat distances (19 kbp and 28.4 kbp), BLM either promoted these events or the inhibitory role was markedly diminished compared to the intermediate distances (i.e., 3.3 kbp and/or 9.1 kbp).

We also tested expression of human BLM WT versus a mutant that is disrupted in the ATP-binding site in the helicase domain (K695A/D795A; Rong et al., 2000; Wang et al., 2018b) in siBLM-treated cells. We first examined RMDs with 1% and 3% divergence at the 9-kbp 3′-DSB/repeat distance, which are increased by BLM depletion (Figures 1C and 1D). We found that expression of human BLM WT caused a marked decrease in these events compared to both the control empty vector (EV) and the K695A/D795A mutant (Figure S1B). We also examined RMDs with identical repeats for the 28.4-kbp 3′-DSB/repeat distance, which is decreased by BLM depletion (Figures 1C, 1D, and S1B), and found that expression of human BLM WT and K695A/D795A had no obvious effect (Figure S1B). We suggest that expression of human BLM WT did not cause an increase in these RMDs, because overexpression of BLM causes a shift toward inhibition of RMDs, as shown above for the comparison of $Blm^{tet/tet}$ versus WT (Figure S1A). In summary, these findings support the above conclusion that, for long DSB/repeat distances, BLM either promotes these events or the inhibitory effect is diminished. Furthermore, these findings indicate that BLM-mediated suppression of RMDs requires the helicase domain.

We also performed additional controls with BLM depletion. To test for possible effects on Cas9-induced DSBs, we used the Surveyor nuclease assay to examine mutagenic end joining at a Cas9 target site (Mendez-Dorantes et al., 2018) and found no obvious effect with either approach to deplete BLM (Figure S2A). We also found that BLM depletion by either approach did not affect cell cycle profiles (Figure S2B).

The DSB End Resection Factors EXO1 and DNA2 Are Particularly Important for RMDs with Long 3′-DSB/Repeat Distances, Similar to BLM

The above findings indicate that RMDs at long 3′-DSB/repeat distances can be promoted by BLM, which we posited might indicate a role for BLM in extensive DSB end resection (Symington and Gautier, 2011). A corollary of this hypothesis is that other factors important for extensive end resection may also be particularly required for such RMDs. To test this hypothesis, we examined EXO1 and DNA2, which were shown in *S. cerevisiae* to have redundant functions in promoting DSB end resection (Gravel et al., 2008; Mimitou and Symington, 2008; Zhu et al., 2008). Other studies have also implicated mammalian EXO1 and DNA2 in DSB end resection (Gravel et al., 2008; Karanja et al., 2012; Nimonkar et al., 2011; Sturzenegger et al., 2014; Tomimatsu et al., 2012).

To examine DNA2, we used a pool of four siRNA-targeting DNA2 (siDNA2), which we confirmed depletes DNA2 mRNA (Figures 2A and 2B). With RMDs between identical repeats, siDNA2

(D) RMD frequencies for WT (EV), $Exo1^{-/-}$ mESCs (EV), and $Exo1^{-/-}$ mESCs transfected with an EXO1 expression vector (EXO1). n = 6. *p $\leq$ 0.0042 and †p-unadjusted = 0.0388 for $Exo1^{-/-}$ versus WT and for $Exo1^{-/-}$ EV versus EXO1 complemented. Fold effects as in (A) are shown.

(E) Effects of EXO1-D173A versus WT on the 28.4-kbp RMD with identical repeats in $Exo1^{-/-}$ mESCs. n = 6. *p < 0.0001 versus $Exo1^{-/-}$ EV. WT EV frequencies are from (D). Fold-effects as in (A) are shown. Immunoblotting analysis of EXO1 and ACTIN using these vectors is shown.

caused a substantial decrease in RMDs for the long 3′-DSB/repeat distances (19 kbp and 28.4 kbp). In contrast, siDNA2 caused a modest reduction for the 9.1 kbp (1.3-fold) and had no effect at the 3.3-kbp and 16-bp 3′-DSB/repeat distances (Figures 2A and 2B). For the 1% divergent RMD events, we found a similar pattern: siDNA2 did not cause a decrease in RMDs for the short/intermediate 3′-DSB/repeat distances (no effect at 16 bp and 9 kb and a modest increase at 3.3 kbp) but caused a significant reduction in RMD frequency for the long distances (1.9-fold for 19 kbp and 2.1-fold for 28.4 kbp). For the 3% divergent RMD events, we found that siDNA2 had no significant effect. Thus, DNA2 appears to promote RMDs between identical and 1% divergent repeats, specifically at long DSB/repeat distances. We also examined expression of 3×FLAG-tagged human DNA2 WT and three mutants deficient in the nuclease, helicase, or both domains (i.e., D277A, K654A, and D277A/K654A, respectively; Figure 2C; Pinto et al., 2016), because these domains are important for DNA replication (Duxin et al., 2012; Li et al., 2018). We examined RMDs between identical repeats at the 28.4-kbp distance, which is reduced with siDNA2 (Figures 2A and 2B). We found that DNA2 WT caused a substantial increase in these RMDs (4-fold), K654A caused a slight increase (1.3-fold), and D277A and D277A/K654A failed to cause an increase versus control EV (expression confirmed by FLAG immunoblot; Figure 2C). Thus, both the DNA2 nuclease and helicase domains appear important for promoting RMDs with a long DSB/repeat distance.

To examine EXO1, we integrated the RMD-GFP reporters in *Exo1*$^{-/-}$ mESCs (Chen et al., 2017) and conducted the RMD assays along with an EXO1 complementation vector or control EV. With RMDs between identical repeats, *Exo1*$^{-/-}$ versus WT mESCs showed a marked reduction in these events for long 3′-DSB/repeat distances (2.8-fold for 19 kbp and 3.8-fold for 28.4 kbp), which was reversed upon complementation with EXO1 (Figure 2D). For the intermediate 3′-DSB/repeat distances, *Exo1*$^{-/-}$ showed reduced frequencies versus WT, but the fold effects were lower than for the long distances (i.e., 1.8-fold for 9.1 kbp and 1.6-fold for 3.3 kbp) and furthermore were not restored by EXO1 complementation. Finally, for 16 bp, RMD frequencies were not distinct between *Exo1*$^{-/-}$ versus WT. For the 1% and 3% RMD reporters, we found that *Exo1*$^{-/-}$ did not show a reduction in RMD frequency at any of the 3′-DSB/repeat distances compared to WT (Figure 2D). We then compared expression of EXO1 WT to a mutant deficient in exonuclease activity (D173A; Orans et al., 2011) for the RMD between identical repeats and the 28.4-kbp distance and found that only EXO1 WT was proficient at promoting this event (expression confirmed by immunoblotting; Figure 2E). These results indicate that EXO1 is required for RMDs between identical repeats at long DSB/repeat distances, similar to DNA2 and BLM, and that EXO1 nuclease activity is important for this function.

The Influence of EXO1 on Promoting RMDs Is Not Epistatic with the Effect of DNA2 and BLM and Is Consistent with a Role in End Resection

In several reports, EXO1 has been shown to have redundant functions with DNA2 and SGS1/BLM for DSB end resection (Gravel et al., 2008; Mimitou and Symington, 2008; Zhu et al., 2008). Thus, we tested whether depletion of DNA2 and BLM might further reduce RMD frequencies in *Exo1*$^{-/-}$ mESCs. We focused on RMDs between identical repeats, as these were the events promoted by these factors, particularly for the long 3′-DSB/repeat distances (Figures 1C, 1D, 2A, and 2D). We confirmed that treatment of siDNA2 and siBLM in *Exo1*$^{-/-}$ mESCs caused depletion of DNA2 mRNA and BLM protein (Figure 3A). We also determined that such siRNAs and EXO1 loss had no obvious effect on cell cycle profiles versus WT (Figure S2B). We found that siDNA2 in *Exo1*$^{-/-}$ mESCs caused a decrease in RMDs at all 3′-DSB/repeat distances (Figure 3B). This effect was modest at 16-bp and 3.3-kbp 3′-DSB/repeat distances (1.3-fold or 1.6-fold, respectively) and more substantial ≥9.1 kbp (i.e., ≥2.7-fold). For example, for the 19-kbp distance, combined loss of DNA2 and EXO1 caused an 18-fold reduction in RMD frequency compared to WT (Figure 3B). We then examined BLM and found that siBLM in *Exo1*$^{-/-}$ mESCs also led to a marked reduction in RMD frequency for the long 3′-DSB/repeat distances (4.4-fold at 19 kbp and 3.3-fold at 28.4 kbp; Figure 3B). Thus, for the long 3′-DSB/repeat distances, combined loss of EXO1 and BLM also caused a drastic reduction in RMD frequency compared to WT (>10-fold; Figure 3B). Furthermore, with *Exo1*$^{-/-}$ mESCs treated with siBLM, expression of human BLM WT caused a significant increase in RMDs with the 28.4-kbp 3′-DSB/repeat distance (Figure S1B). In comparing the helicase-deficient mutant (K695A/D795A) versus BLM WT in this experiment, the RMD frequency for the mutant was slightly lower, but not statistically different (Figure S1B). For the shorter 3′-DSB/repeat distances, siBLM treatment in *Exo1*$^{-/-}$ mESCs caused no significant effects at the 3′-DSB/repeat distances of 3.3 kbp and 16 bp and a modest (1.5-fold) decrease at 9.1 kbp (Figure 3B). In summary, combined disruption of DNA2 and EXO1, and BLM and EXO1, causes a drastic reduction in RMDs at long DSB/repeat distances.

We also examined CtIP, which is important for initiation of end resection (Sartori et al., 2007), by depleting CtIP alone (siCtIP) and in combination with siBLM. Similar to the findings in the prior study with the RMD-GFP reporter (Mendez-Dorantes et al., 2018), we found that siCtIP caused a marked decrease in RMDs at all 3′-DSB/repeat distances (≥3.1-fold; Figure S3). Thus, CtIP-mediated initiation of end resection is important for RMD events irrespective of 3′-DSB/repeat distances. Adding siBLM with siCtIP caused a significant increase in RMDs at the intermediate distances (3.3 kbp and 9.1 kbp), and a modest decrease at the 28.4-kbp distance, as compared to siCtIP alone (Figure S3). This finding supports the notion that BLM and CtIP have distinct effects on RMDs.

We sought to contrast our findings with RMD events versus detection of end resection via a flow-cytometry-based assay for chromatin-bound RPA (an ssDNA binding factor), which also includes staining with the DNA dye DAPI to examine cell cycle phase (Forment et al., 2012). To induce DSBs, we used the topoisomerase II poison etoposide (Nitiss, 2009), which we found caused a significant increase in WT mESCs with high chromatin-bound RPA (Figure 3C). For *Exo1*$^{-/-}$, and WT treated with siDNA2, we observed a significant reduction in such RPA staining after etoposide treatment compared to WT siCTRL (Figure 3C). We found that siDNA2 treatment of *Exo1*$^{-/-}$ cells did

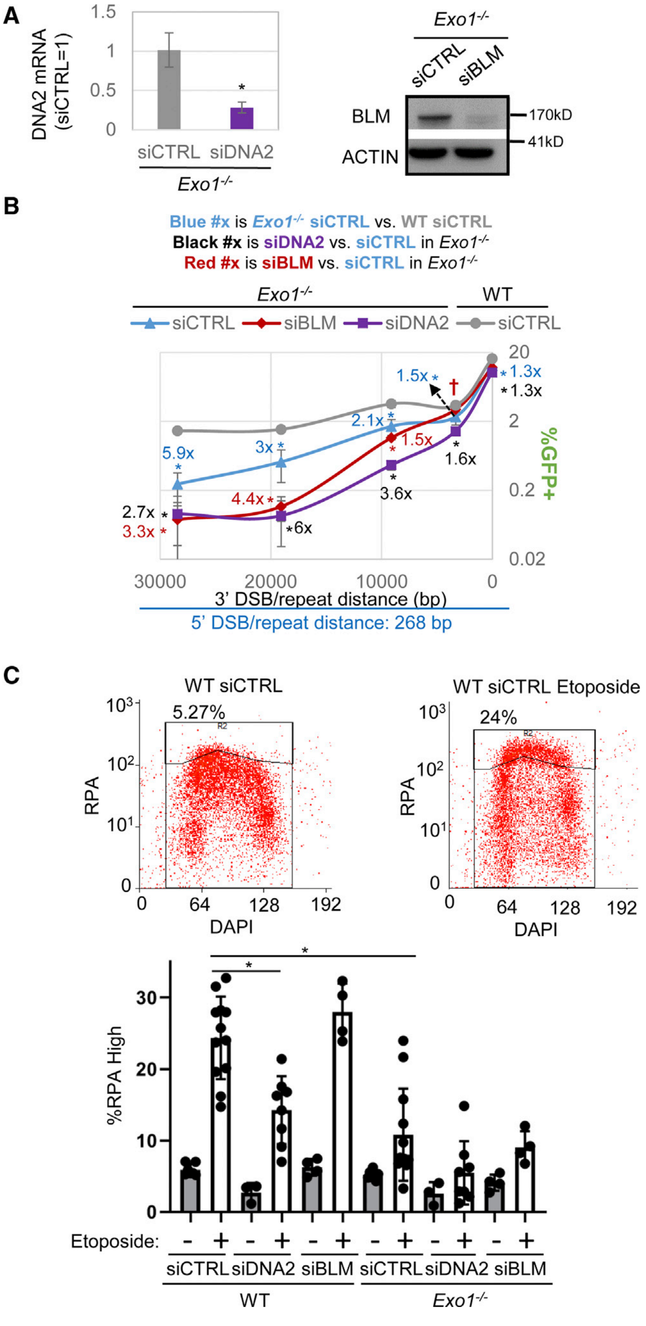

Figure 3. The Influence of EXO1 on Promoting RMDs Is Not Epistatic with the Effect of DNA2 and BLM and Is Consistent with a Role in End Resection

(A) RT-PCR of DNA2 mRNA and immunoblotting analysis of BLM and ACTIN of *Exo1*$^{-/-}$ mESCs treated with the respective siRNAs. n = 3 PCR. *p = 0.052.

(B) RMD analysis of *Exo1*$^{-/-}$ mESCs treated with siCTRL, siDNA2, or siBLM along with WT (siCTRL). n = 6. *p $\leq$ 0.0179 *Exo1*$^{-/-}$ siCTRL versus siDNA2 and *Exo1*$^{-/-}$ siCTRL versus WT siCTRL. *p $\leq$ 0.0435 and †p-unadjusted = 0.0274 for *Exo1*−/− siCTRL versus siBLM. Shown are fold effects of significant differences, using the mean.

(C) Influence of DNA2, BLM, and EXO1 on etoposide-induced chromatin-bound RPA. Cells were incubated with 3 μM etoposide or DMSO control, detergent extracted, and stained for RPA and DAPI. Shown are representative flow cytometry plots with the upper gate showing high RPA staining (upper) and percentages of cells with such staining (lower). n = 3 for DMSO. For etoposide treated, n = 12 for siCTRL, n = 8 for siDNA2, and n = 4 for siBLM. *p < 0.001.

not have an obvious effect; however, RPA staining in *Exo1*$^{-/-}$ cells treated with etoposide was near background levels (Figure 3C). Finally, siBLM-treated cells were not distinct from siCTRL for both WT and *Exo1*$^{-/-}$ (etoposide treated cells; Figure 3C). These findings support a role for EXO1 and DNA2 in DSB end resection in mESCs. In contrast, the roles of BLM in DSB end resection were not readily revealed by this assay, which may reflect an anti-recombination activity of BLM that could stabilize RPA-bound ssDNA.

BLM-Mediated Suppression of RMDs Is Not Epistatic with the Influence of MSH2 (except at the 16-bp Distance) or BRCA2

We next sought to compare BLM to another factor that inhibits RMDs between divergent repeats: the mismatch repair factor MSH2 (Mendez-Dorantes et al., 2018). In particular, we tested whether BLM and MSH2 are epistatic for inhibiting such RMDs, using siBLM treatment in *Msh2*$^{-/-}$ mESCs, which we confirmed causes a reduction in BLM protein levels (Figure 4A). For RMDs with identical repeats, we compared *Msh2*$^{-/-}$ mESCs to WT and found that loss of MSH2 affected RMD frequencies for the 3′-DSB/repeat distances of 3.3 and 28.4 kbp (2.1-fold higher and 1.8-fold lower, respectively) but had no effect at the other distances (Figure 4A). We then examined the effect of siBLM in *Msh2*$^{-/-}$ mESCs and found similar results to siBLM in WT, i.e., a marked reduction in RMDs for long 3′-DSB/repeat distances (4.9-fold for 19 kbp and 7.7-fold for 28.4 kbp; Figure 4A). Also in *Msh2*$^{-/-}$ mESCs, siBLM caused modest effects at 16 bp and 3.3 kbp (1.5-fold lower and 1.1-fold higher, respectively) and no effect at 9.1 kbp (Figure 4A).

For the 1% and 3% divergent repeats, we found that *Msh2*$^{-/-}$ mESCs showed a marked increase in RMD frequency at all 3′-DSB/repeat distances compared to WT ($\geq$4-fold), as expected (Hu et al., 2019; Mendez-Dorantes et al., 2018). Strikingly, we found that siBLM in *Msh2*$^{-/-}$ mESCs caused a further increase in RMD frequency between 1% or 3% divergent repeats at 3.3 kbp and 9.1 kbp ($\geq$3.1-fold; Figure 4A). Thus, combined loss of MSH2 and BLM causes a dramatic increase in RMDs between divergent repeats at these intermediate 3′-DSB/repeat distances ($\geq$21-fold; Figure 4A). We then examined the long 3′-DSB/repeat distances. We found that, for the 1% divergent repeats, siBLM treatment in *Msh2*$^{-/-}$ mESCs caused a decrease in RMDs (1.5-fold for 19 kbp and 2.2-fold for 28.4 kbp; Figure 4A). For the 3% divergent repeats, siBLM caused a modest increase at 19 kbp (1.4-fold) and had no effect at 28.4 kbp (Figure 4A). Importantly, siBLM treatment in *Msh2*$^{-/-}$ mESCs did not have a substantial effect on RMDs with the short 3′-DSB/repeat distance (16 bp; Figure 4A), which is distinct from the effect of siBLM in WT cells (i.e., caused an increase for the divergent repeats; Figure 1C). These findings indicate that BLM and MSH2 are not epistatic for inhibiting RMDs between divergent repeats with intermediate 3′-DSB/repeat distances (i.e., 3.3 kbp and 9.1 kbp) but are epistatic for the short distance

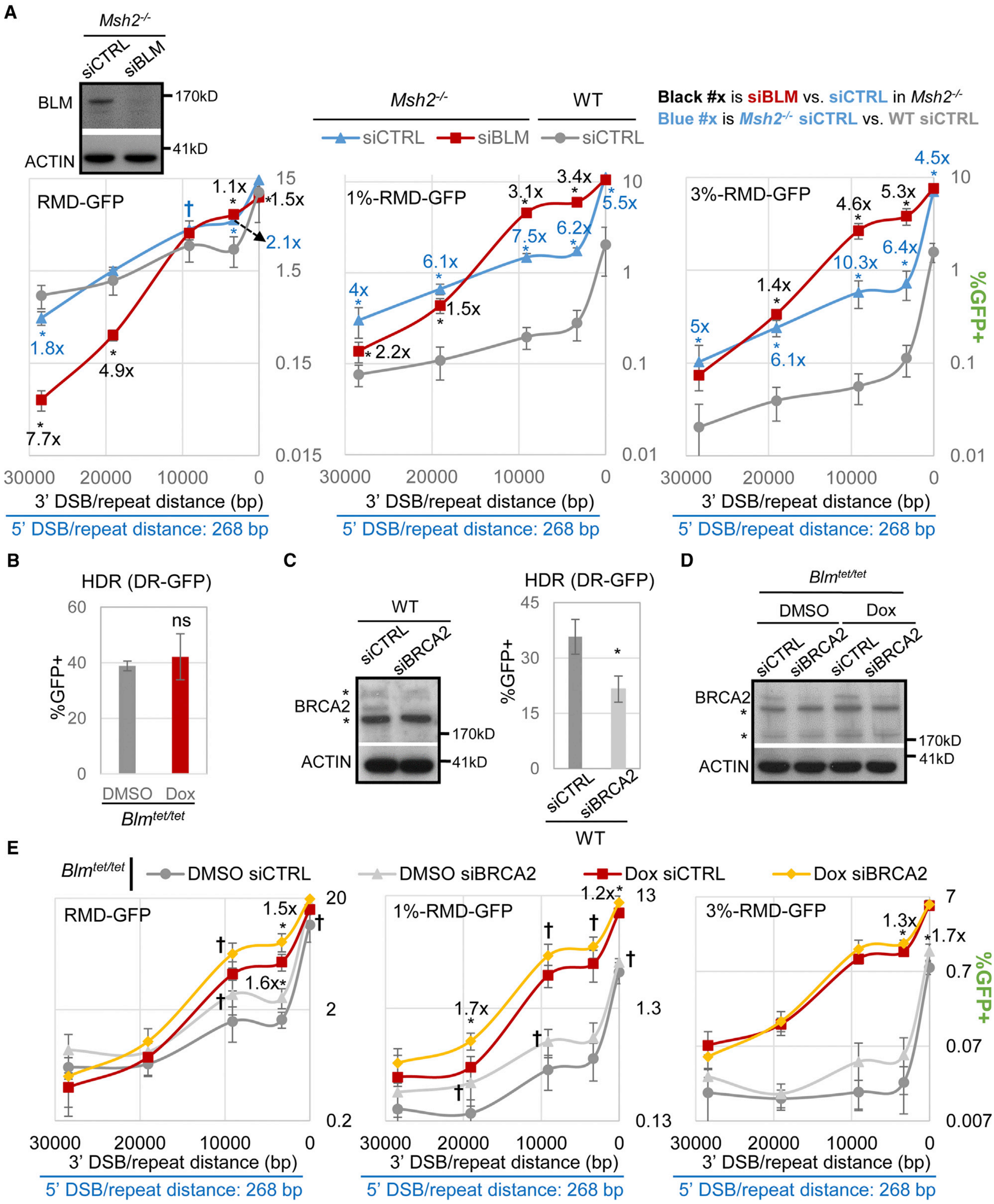

Figure 4. BLM-Mediated Suppression of RMDs Is Not Epistatic with the Influence of MSH2 (except at the 16-bp Distance) or BRCA2

(A) Influence of BLM depletion on RMDs in *Msh2*$^{-/-}$ mESCs. Shown is BLM and ACTIN immunoblot analysis, and RMD frequencies, of *Msh2*$^{-/-}$ mESCs treated with siBLM versus siCTRL. The WT siCTRL RMD frequencies are from Figure 1C. n = 6. *p $\leq$ 0.0222 and †p-unadjusted $\leq$ 0.0272 for siCTRL versus siBLM in *Msh2*$^{-/-}$ mESCs or for WT versus *Msh2*$^{-/-}$. Shown are fold effects of significant differences, using the mean.

(legend continued on next page)

(16 bp). Furthermore, in MSH2-deficient cells, BLM promotes RMDs with long 3′-DSB/repeat distances, as in WT cells.

We also sought to contrast the role of BLM in these RMD events with another homologous recombination pathway, homology-directed repair (HDR), using the DR-GFP reporter (Moynahan et al., 2001). For these experiments, we induced the DSB in this reporter using Cas9 and a sgRNA (Muñoz et al., 2014), to be consistent with the RMD analysis. We used the *Blm*$^{tet/tet}$ cell line with the DR-GFP reporter targeted to the *Pim1* locus and found that BLM depletion did not obviously affect the frequency of HDR (Figure 4B), similar to prior findings with another HDR assay (Larocque and Jasin, 2010). In contrast, depletion of BRCA2 via a pool of four siRNAs (siBRCA2) caused a significant reduction in this HDR event (Figure 4C), as expected (Moynahan et al., 2001).

We then examined the influence of BRCA2 on RMDs in BLM-proficient and deficient cells. For this, we treated the *Blm*$^{tet/tet}$ RMD reporter cell lines with siBRCA2, which we confirmed causes depletion of BRCA2 protein (Figure 4D). We found that siBRCA2 depletion caused an increase in RMDs between identical repeats at the intermediate 3′-DSB/repeat distances (3.3 kbp and 9.1 kbp; the latter statistically significant only without multiple comparison correction) both in the Dox-treated and control (DMSO-treated) cells (Figure 4E). Similarly, siBRCA2 treatment caused an increase in RMDs for the 1% divergent repeats at several 3′-DSB/repeat distances in both the Dox-treated and control cells (Figure 4E; although some are significant only without multiple comparison correction). For the 3% divergent repeats, siBRCA2 had minimal effects (Figure 4E). Altogether, these findings indicate that BRCA2 can suppress a subset of RMDs, which is consistent with prior findings (Mendez-Dorantes et al., 2018; Stark et al., 2004; Tutt et al., 2001). Furthermore, the influence of BRCA2 appears independent of BLM.

RAD52 Substantially Promotes a Subset of RMDs; however, BLM and MSH2 Inhibit RMDs Independently of RAD52

We next sought to examine the interplay between BLM and RAD52 on RMD formation. RAD52 is a DNA binding protein that can mediate annealing between homologous sequences (Brouwer et al., 2017; Grimme et al., 2010). Previously, using a *Rad52*$^{-/-}$ mESC line, our laboratory showed that RAD52 promotes RMDs between identical repeats at 3′-DSB/repeat distances of $\geq$3.3 kbp but does not promote such RMDs at the 16-bp distance (Mendez-Dorantes et al., 2018). We confirmed these findings by comparing *Rad52*$^{-/-}$ cells with both WT and *Rad52*$^{-/-}$ cells transfected with a RAD52 expression vector, which causes overexpression of RAD52, as well as higher RMD frequencies than WT (Figures 5A and 5B). We then examined RMDs with 1% divergent repeats and found similar results, in that RAD52 is markedly required for such RMDs at 3′-DSB/repeat distances of $\geq$3.3 kbp (Figure 5B). For the 3% divergent repeats, loss of RAD52 caused a significant decrease for the 3.3-kbp and 9.1-kbp distances (the former only when not adjusted for multiple comparisons; Figure 5B). Also, RAD52 expression in the *Rad52*$^{-/-}$ mESCs caused a striking increase in RMDs with 3% divergent repeats for 3′-DSB/repeat distances $\geq$3.3 kbp (Figure 5B). For the 16-bp 3′-DSB/repeat distance, we found that loss of RAD52 failed to cause a reduction of RMDs between identical or divergent repeats (Figure 5B). Thus, RAD52 is critical for RMDs with 3′-DSB/repeat distances $\geq$3.3 kbp, but its requirement appears diminished with 3% divergent repeats, although overexpression of RAD52 can promote these events.

We then tested whether the role of RAD52 in RMDs is epistatic with BLM and/or MSH2. As one possibility, loss of BLM and/or MSH2 might fail to cause an increase in RMDs in *Rad52*$^{-/-}$ mESCs. These findings would indicate that BLM and/or MSH2 specifically disrupt the RAD52 annealing intermediate. Alternatively, loss of BLM and/or MSH2 might cause an increase in RMDs in *Rad52*$^{-/-}$ mESCs. Such findings would indicate that suppression of RMDs via BLM and/or MSH2 is not specific to RAD52 annealing intermediates.

Thus, we examined the effects of siBLM in *Rad52*$^{-/-}$ mESCs, which we confirmed caused depletion of BLM (Figure 5A). From these experiments, we found that siBLM treatment of *Rad52*$^{-/-}$ mESCs caused a drastic increase in RMDs between 1% and 3% divergent repeats for the 3.3-kbp and 9.1-kbp 3′-DSB/repeat distances ($\geq$6.8-fold; Figure 5C). We also found that siBLM treatment in *Rad52*$^{-/-}$ mESCs caused a significant but less dramatic increase for RMD events with identical repeats for the 3.3-kbp and 9.1-kbp 3′-DSB/repeat distances ($\geq$2-fold) and for divergent repeats for the 16-bp distance ($\leq$2-fold; Figure 5C). Thus, the role of BLM for inhibiting RMDs appears independent of RAD52. For RMDs with long 3′-DSB/repeat distances and identical repeats, whereas siBLM treatment of WT cells caused a reduction in these events (Figure 1C), siBLM treatment of *Rad52*$^{-/-}$ mESCs failed to cause such a decrease (Figure 5C). However, these RMD events in *Rad52*$^{-/-}$ mESCs are near background levels (i.e., $\geq$17-fold lower versus WT).

We then performed analogous experiments with MSH2 and RAD52, using a *Rad52*$^{-/-}$*Msh2*$-/-$ mESC line that we generated (Figure 5A), and determined that loss of MSH2 and/or RAD52 did not obviously affect cell cycle profiles (Figure S2B). For the RMD analysis, an MSH2 expression vector was used, which we confirmed expresses MSH2 (Figure 5A). We found similar effects of MSH2 when comparing either *Rad52*$-/-$*Msh2*$^{-/-}$ versus *Rad52*$^{-/-}$ or *Rad52*$^{-/-}$*Msh2*$^{-/-}$ versus the MSH2 complemented (Figure 5D). For the 1% and 3% divergent repeats, we found that loss of MSH2 in RAD52-deficient cells caused a marked increase in RMDs at all 3′-DSB/repeat distances (Figure 5D). Indeed, we found that RMD frequencies were restored to WT levels for 1% divergent repeats in

(B) Influence of BLM levels on HDR (DR-GFP assay) in *Blm*$^{tet/tet}$ mESCs treated with DMSO or Dox, as for the RMD assays. n = 6. ns, not significant.
(C) Influence of siBRCA2 treatment on HDR in mESCs. n = 6. *p = 0.0002. Immunoblot analysis of BRCA2 and ACTIN using this siRNA is shown. The asterisk (*) indicates nonspecific bands.
(D) Immunoblot analysis of BRCA2 depletion in *Blm*$^{tet/tet}$ mESCs treated with DMSO or Dox.
(E) Effects of siBRCA2 on RMDs in *Blm*$^{tet/tet}$ mESCs treated with Dox (i.e., BLM depletion) or DMSO control. n = 6. *p $\leq$ 0.0354 and †p-unadjusted $\leq$ 0.0435 for siCTRL versus siBRCA2. Fold effects as in (A) are shown.

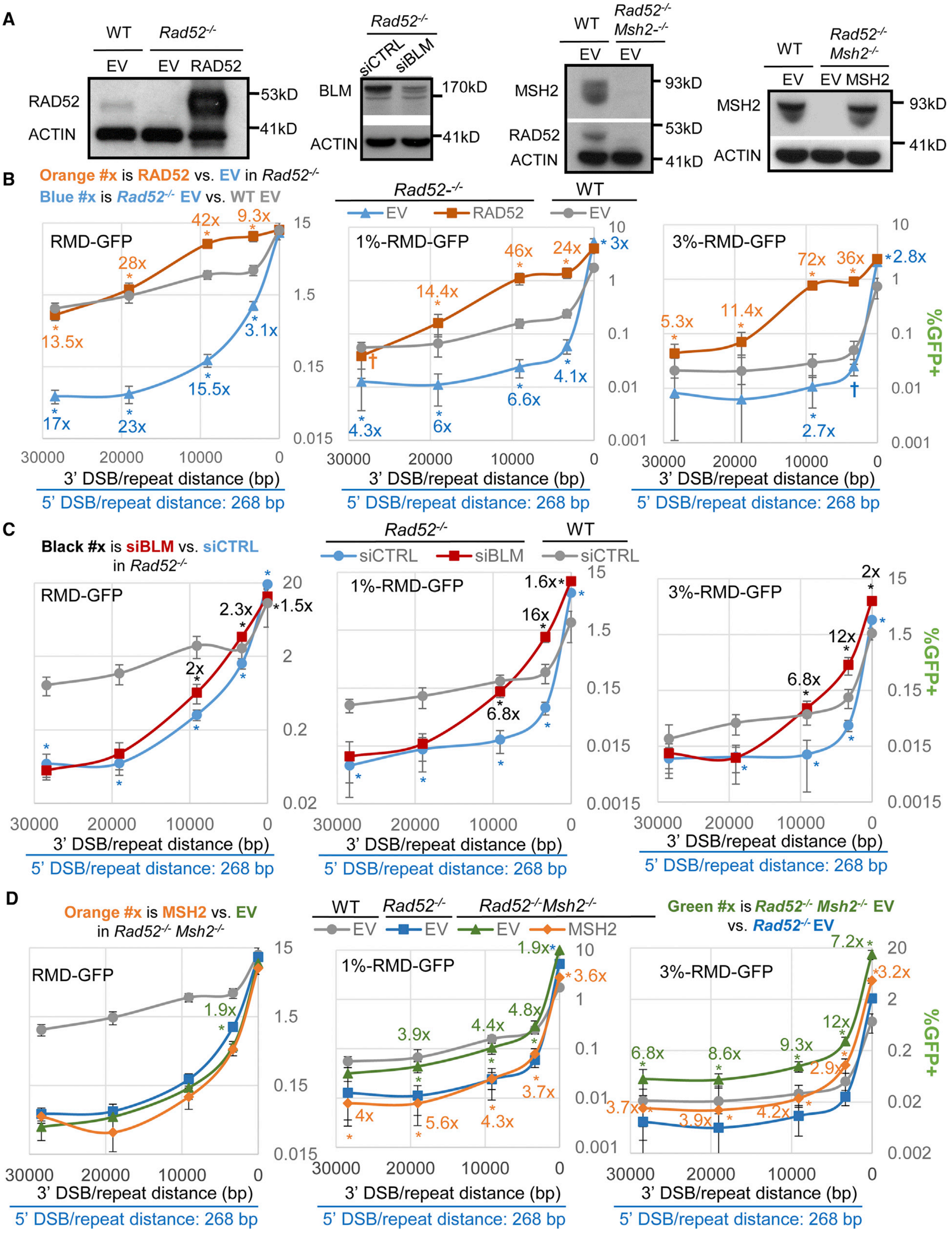

(legend on next page)

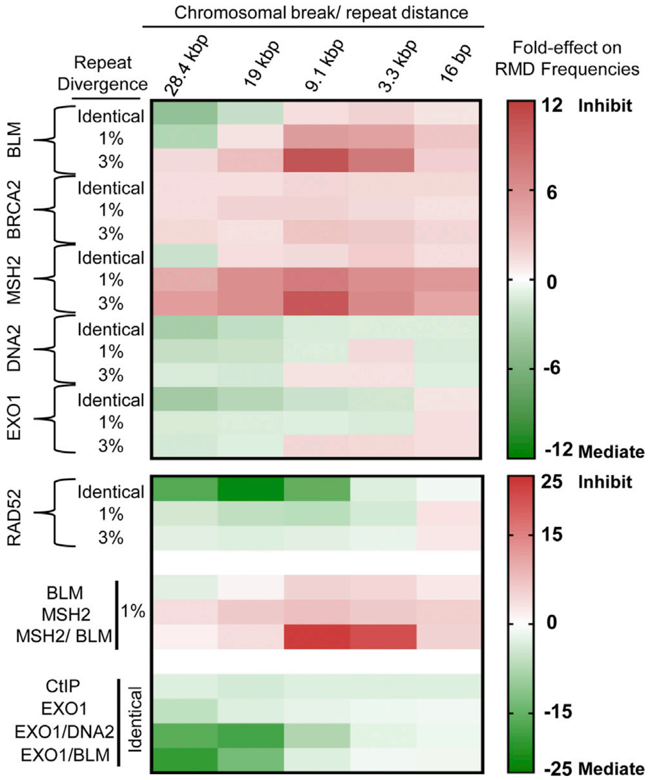

Figure 6. Summary of Effects of Individual Factors on RMDs

Shown are two heatmaps with the fold effects of individual factors on RMDs (versus the WT control) at distinct DSB/repeat distances and between identical and divergent repeats. The BLM fold effects shown are siBLM versus siCTRL.

Rad52$^{-/-}$*Msh2*$^{-/-}$ mESCs and were even higher than WT levels for 3% divergent repeats in *Rad52*$^{-/-}$*Msh2*$^{-/-}$ mESCs (Figure 5D). For the identical repeats, loss of MSH2 caused a 1.9-fold reduction in the RMD frequency for the 3.3-kbp distance but had no effect at the other distances (Figure 5D). In summary, both MSH2 and BLM appear to inhibit RMDs independently of RAD52.

DISCUSSION

To understand the mechanism of RMD formation, we have examined the relative role of several factors on RMDs under varying conditions of DSB/repeat distance and sequence divergence. We found that these specific conditions can have a striking impact on the influence of individual factors on RMDs, which we have summarized in a heatmap (Figure 6). In particular, we found that the BLM helicase has contrary effects on RMDs, depending on DSB/repeat distance and sequence divergence. For intermediate DSB/repeat distances and divergent repeats, BLM markedly suppresses RMDs. In contrast, for long DSB/repeat distances, BLM either promotes these events or the inhibitory effect is markedly diminished. Finally, for short DSB/repeat distances, the influence of BLM on RMDs is diminished, including for events with sequence divergence. Based on the SSA model for RMDs, we suggest that BLM promotes DSB end resection, although this role is only evident for events with long DSB/repeat distances. We also suggest that BLM promotes heteroduplex rejection; however, this role is most substantial for events with intermediate DSB/repeat distances.

A role for BLM during end resection is supported by experiments with purified proteins (Daley et al., 2017; Nimonkar et al., 2011; Soniat et al., 2019), but we did not observe an obvious effect on damage-induced chromatin-bound RPA, which is consistent with another study (Patel et al., 2017). These results may reflect the contrary roles of BLM during homologous recombination in that, by promoting heteroduplex rejection, BLM could stabilize RPA-bound end resection intermediates. In contrast, the nucleases EXO1 and DNA2, which do not appear to have an inhibitory role for RMDs, appear important for both RMDs with long DSB/repeat distances as well as end resection (i.e., damage-induced chromatin-bound RPA), although, for RMDs with sequence divergence, we found that the requirement for DNA2 and EXO1 is diminished compared to identical repeats. The reason for this distinction is unclear, but we suggest that the rate-limiting step of RMDs with sequence divergence is evading heteroduplex rejection. Namely, although loss of EXO1 and DNA2 likely causes slow kinetics of extensive end resection, evading heteroduplex rejection could be much slower and/or infrequent, such that the influence of EXO1 and DNA2 on these events would be diminished.

Regarding specific requirements for BLM, EXO1, and DNA2, each of these factors are particularly important for RMDs with DSB/repeat distances that are $\geq$19 kbp. We also performed double mutant analysis and found that disrupting both EXO1 with DNA2 caused a marked reduction in RMDs at the 9.1-kbp distance. In contrast, these factors are largely dispensable for RMDs with the 16-bp and 3.3-kbp DSB/repeat lengths. These findings are consistent with a dispensable role of DNA2 for measurements of end resection between approximately 0.3–3.5 kbp, although such assays based on AsiSI-induced DSBs have not been shown to detect resection beyond this range (Myler et al., 2016; Zhou et al., 2014). In contrast, EXO1 is important 0.3–3.5 kbp of end resection, based on this assay (Zhou et al., 2014). Nonetheless, EXO1 has been shown to be dispensable for homologous recombination events requiring this amount of end resection (i.e., <3 kbp; Chen et al., 2017; Rein et al., 2015).

Figure 5. RAD52 Substantially Promotes a Subset of RMDs; however, BLM and MSH2 Inhibit RMDs Independently of RAD52

(A) Immunoblotting analysis for several mESC lines, and transfections of siRNA or complementation vectors, for RAD52, MSH2, BLM, and ACTIN.

(B) RMD analysis in WT (EV), *Rad52*$^{-/-}$ (EV), and *Rad52*$^{-/-}$ mESCs transfected with a RAD52 expression vector. Frequencies for WT EV are from Figure 2D. n = 6. *p $\leq$ 0.0481 and †p-unadjusted $\leq$ 0.0332 for EV versus RAD52 complemented in *Rad52*$^{-/-}$ or WT versus *Rad52*$^{-/-}$.

(C) RMD frequencies of *Rad52*$^{-/-}$ mESCs treated with siBLM or siCTRL. Frequencies from WT siCTRL are from Figure 1C. n = 6. *p $\leq$ 0.0036 for siCTRL versus siBLM or for WT versus *Rad52*$^{-/-}$.

(D) RMD analysis for WT, *Rad52*$^{-/-}$ mESCs, *Rad52*$^{-/-}$*Msh2*$^{-/-}$, and *Rad52*$^{-/-}$*Msh2*$^{-/-}$ transfected with an MSH2 expression vector. Frequencies for WT EV and *Rad52*$^{-/-}$ EV are from Figure 2D and (B), respectively. n = 6. *p $\leq$ 0.0221 for *Rad52*$^{-/-}$ versus *Rad52*$^{-/-}$*Msh2*$^{-/-}$ or for MSH2 complemented versus EV.

We speculate that, in cells deficient in EXO1 and DNA2, other nucleases can mediate end resection until approximately 9 kbp. Nevertheless, end resection <9 kb likely occurs at a reduced efficiency and/or rate in cells without EXO1 and DNA2, because end resection as measured by chromatin-bound RPA and/or the AsiSI nuclease assays is reduced in such cells. Alternatively, it is formally possible that such recombination events may have a resection-independent mechanism. This latter possibility may be unlikely, because RMDs at all DSB/repeat lengths are dependent on the end resection initiation factor CtIP (Daley et al., 2017; Deshpande et al., 2016; Makharashvili and Paull, 2015; Sartori et al., 2007). In summary, BLM, EXO1, and DNA2 in mammalian cells appear particularly important for homologous recombination events (e.g., RMD events) requiring end resection that is $\geq$9.1 kbp, as compared to $\leq$3.3 kbp.

Regarding physiological significance, such extensive resection to enable RMDs may be required under conditions where resection has initiated but HDR is not feasible, such as in the absence of the sister chromatid template (Johnson and Jasin, 2000). Because such resected ends also may not be readily repaired by end joining, RMDs may be required to ensure restoration of the chromosome at the expense of a deletion mutation between the repeats. Accordingly, facilitating RMDs is an example of cells ensuring survival at the cost of loss of genetic information (Krenning et al., 2019; Nik-Zainal and Hall, 2019).

Considering the mechanism of such resection, both the helicase and nuclease domains of DNA2 appear important for RMDs with a long DSB/repeat distance. As one possibility, the DNA2 helicase domain could be important to unwind complex DNA structures (e.g., G-quadruplex) during end resection, as is proposed for its role in DNA replication (Duxin et al., 2012; Li et al., 2018; Masuda-Sasa et al., 2008; Thangavel et al., 2015). However, we examined the sequence of the RMD reporter for possible G-quadruplex-forming repeats (Cer et al., 2013) and did not observe an obvious enrichment of such sequences far from the repeat, or of the repeats themselves (identical or divergent), but rather found a set of such sequences just upstream of the repeat (Figure S4). As an alternative mechanism, we suggest that the helicase domain of DNA2 may be important for unwinding long stretches of DNA to facilitate nucleolytic degradation by its nuclease domain and/or EXO1, which is supported by studies of the DNA2 helicase activity (Pinto et al., 2016).

DSB/repeat distance not only affects the amount of end resection required for RMDs but also the length of the 3′ ssDNA that flanks the annealing intermediate (i.e., the length of the non-homologous tail). In *S. cerevisiae*, heteroduplex rejection has been shown to require such a non-homologous tail (Anand et al., 2017; Chakraborty and Alani, 2016; Chakraborty et al., 2016; Haber, 2018). The precise influence of the non-homologous tail on heteroduplex rejection is unclear. One proposed model is that the MSH2/MSH6 complex preferentially recruits the SGS1 helicase to heteroduplexes with a non-homologous tail (Anand et al., 2017; Chakraborty and Alani, 2016; Chakraborty et al., 2016; Haber, 2018). In contrast, without a non-homologous tail, SGS1 may not be recruited and/or may not unwind such substrates (Chakraborty et al., 2016). Such MSH2-mediated heteroduplex rejection in *S. cerevisiae* requires only a very short non-homologous tail ($\sim$30 bp; Anand et al., 2017), which is consistent with our findings that MSH2 suppresses RMDs between divergent repeats for all DSB/repeat distances.

However, for the BLM helicase, suppression of RMDs between divergent repeats is most predominant for intermediate DSB/repeat distances, indicating that BLM-mediated heteroduplex rejection functions through different parameters than MSH2. Indeed, the BLM-mediated suppression of these RMDs is not epistatic with MSH2, indicating that BLM can disrupt heteroduplex DNA independently of MSH2. We also found that the BLM helicase domain is required for inhibition of such RMDs, which is consistent with a direct role in unwinding heteroduplex DNA. This finding is not necessarily expected, because several anti-recombination functions of *SGS1* in *S. cerevisiae* have been shown to be independent of its helicase domain (Jain et al., 2009; Lo et al., 2006; Piazza et al., 2019; Weinstein and Rothstein, 2008). BLM-mediated unwinding of heteroduplex DNA appears to be optimal for events with a long non-homologous tail, given that BLM caused the greatest suppression of RMDs with intermediate DSB/repeat distances ($\geq$3.3 kbp). As one possible mechanism, cleavage and/or degradation of a long non-homologous tail may have slower kinetics than a shorter tail and hence may be more prone to unwinding via BLM. Alternatively, a long non-homologous tail may bind a specific factor(s), or form a particular secondary structure, that recruits BLM to the heteroduplex DNA.

The physiological significance of a long non-homologous tail is that this structure is likely a key indicator of aberrant recombination intermediates. Namely, nonallelic homologous recombination (NAHR) likely involves divergent sequences that are also flanked by a non-homologous tail (Carvalho and Lupski, 2016). In contrast, this tail is absent in recombination between sister chromatids. Similarly, allelic recombination between homologous chromosomes may have sequence divergence but is unlikely to involve a long non-homologous tail. Thus, requiring both sequence divergence and a long non-homologous tail for optimal BLM function may ensure that BLM inhibits NAHR while allowing for sister chromatid and allelic recombination. Consistent with this notion, BLM does not appear to have an obvious suppressive effect on allelic recombination, apart from inhibiting crossover products (LaRocque et al., 2011). Finally, although we propose that the major effects of BLM on RMDs is via regulation of DSB end resection and heteroduplex rejection, it is also possible that BLM could affect the synapsis of DSB ends across long distances and/or the induction or persistence of Cas9 DSBs, although, regarding the latter, BLM depletion did not have an obvious effect on Cas9-induced mutagenic end joining.

In addition to BLM, we also found that DSB/repeat distance and repeat divergence affects the relative role of the annealing factor RAD52 during RMDs. As shown previously for RMDs between identical repeats (Mendez-Dorantes et al., 2018), we found that RAD52 is dispensable for these events with short DSB/repeat distances (i.e., 16 bp) but promotes such events with longer distances ($\geq$3.3 kbp). We found similar results with the 1% divergent repeats. As described above, 3′-DSB/repeat distances $\geq$3.3 kbp likely result in an annealing intermediate with a long non-homologous tail. Thus, RAD52 appears particularly important for RMDs that require removal of a long non-homologous tail, which is consistent with recent studies

(Kelso et al., 2019; Wang et al., 2018a). As discussed above in regards to BLM function, removal of a long non-homologous tail may have slower kinetics than for a short tail. Thus, RMDs with a long non-homologous tail may require a highly stable annealing intermediate that is dependent on RAD52. This model is supported by the capacity for RAD52 to stabilize dsDNA, as detected as resistance to force-induced strand separation (Brouwer et al., 2017).

A corollary of this model is that other annealing factors can substitute for RAD52 for RMDs with a short non-homologous tail. Furthermore, such annealing factors also appear to substitute for RAD52 for events with long non-homologous tails but only under specific circumstances. Namely, for 1% divergent repeats and 3′-DSB/repeat distance of 3.3–9.1 kbp, RAD52 is critical for these RMDs but only in the presence of BLM and MSH2. These findings are significant, because inhibitors of RAD52 are being developed for synthetic lethal strategies (Hengel et al., 2017). Namely, our findings raise the notion that such inhibitors may fail to inhibit RMD events in MSH2-deficient tumors, which appear to include both inherited colorectal cancer (Fishel et al., 1993) and such tumors that downregulate mismatch repair in response to therapy (Russo et al., 2019). In addition, RAD52 is dispensable for RMDs with 3% divergent repeats, except for the 9.1-kbp DSB/repeat distance, although overexpression of RAD52 can substantially promote these events for the ≥3.3-kbp distances. Consistent with this notion, a report in human cells found that RMD events between ≥5% divergent repeats are independent of RAD52 (Morales et al., 2015).

Altogether, we suggest that RAD52 is particularly important to mediate annealing of identical and 1% divergent repeats that are flanked by a long non-homologous tail. In contrast, for events involving a short non-homologous tail, 3% sequence divergence, and/or occurring in the absence of heteroduplex rejection (i.e., with loss of BLM or MSH2), other annealing factors appear to substitute for RAD52 to mediate these events. The pathways that mediate such synapsis during RMDs are unclear, although they are unlikely to involve BRCA2, which we find suppresses RMDs, similar to previous studies (Mendez-Dorantes et al., 2018; Stark et al., 2004; Tutt et al., 2001). In conclusion, we suggest that DSB/repeat distance has a substantial influence on the mechanism of RMDs, because this distance affects both the amount of end resection required as well as the length of the non-homologous tail in the annealing intermediate, which appears critical for optimal BLM-mediated heteroduplex rejection.

STAR★METHODS

Detailed methods are provided in the online version of this paper and include the following:

- KEY RESOURCES TABLE
- LEAD CONTACT AND MATERIALS AVAILABILITY
- EXPERIMENTAL MODEL AND SUBJECT DETAILS
- METHOD DETAILS
  - Reporter integration and recombinant DNA
  - RMD and HDR reporter assays
  - End resection assay
  - Immunoblotting and RT-PCR
  - Cell cycle analysis, Surveyor nuclease assay, and G-quandruplex predictions
- QUANTIFICATION AND STATISTICAL ANALYSIS
- DATA AND CODE AVAILABILITY

SUPPLEMENTAL INFORMATION

Supplemental Information can be found online at https://doi.org/10.1016/j.celrep.2020.01.001.

ACKNOWLEDGMENTS

We thank Junji Takeda (Osaka University) for the *BLM*$^{tet/tet}$ mESC line, Maria Jasin (Memorial Sloan Kettering) and Winfried Edelmann (Einstein) for the *Exo1*$^{-/-}$ mESC line, and Samuel Bunting (Rutgers) for technical advice. This study was supported in part by NIH grants R01CA197506 (J.M.S.) and P30CA033572 (City of Hope Core Facilities) and Ford Foundation Predoctoral Fellowship (C.M.-D.).

AUTHOR CONTRIBUTIONS

All authors developed reagents and performed experiments. C.M.-D., L.J.T., and J.M.S. designed experiments and wrote the paper, with input on the editing from all authors.

DECLARATION OF INTERESTS

The authors declare no competing interests.

Received: September 3, 2019
Revised: December 8, 2019
Accepted: December 31, 2019
Published: February 4, 2020

REFERENCES

Ade, C., Roy-Engel, A.M., and Deininger, P.L. (2013). Alu elements: an intrinsic source of human genome instability. Curr. Opin. Virol. *3*, 639–645.

Alani, E., Reenan, R.A., and Kolodner, R.D. (1994). Interaction between mismatch repair and genetic recombination in Saccharomyces cerevisiae. Genetics *137*, 19–39.

Anand, R., Beach, A., Li, K., and Haber, J. (2017). Rad51-mediated double-strand break repair and mismatch correction of divergent substrates. Nature *544*, 377–380.

Bachrati, C.Z., Borts, R.H., and Hickson, I.D. (2006). Mobile D-loops are a preferred substrate for the Bloom's syndrome helicase. Nucleic Acids Res. *34*, 2269–2279.

Batzer, M.A., and Deininger, P.L. (2002). Alu repeats and human genomic diversity. Nat. Rev. Genet. *3*, 370–379.

Bennardo, N., Cheng, A., Huang, N., and Stark, J.M. (2008). Alternative-NHEJ is a mechanistically distinct pathway of mammalian chromosome break repair. PLoS Genet. *4*, e1000110.

Bhargava, R., Onyango, D.O., and Stark, J.M. (2016). Regulation of single-strand annealing and its role in genome maintenance. Trends Genet. *32*, 566–575.

Bond, C.T., Sprengel, R., Bissonnette, J.M., Kaufmann, W.A., Pribnow, D., Neelands, T., Storck, T., Baetscher, M., Jerecic, J., Maylie, J., et al. (2000). Respiration and parturition affected by conditional overexpression of the Ca2+-activated K+ channel subunit, SK3. Science *289*, 1942–1946.

Brouwer, I., Zhang, H., Candelli, A., Normanno, D., Peterman, E.J.G., Wuite, G.J.L., and Modesti, M. (2017). Human RAD52 captures and holds DNA strands, increases DNA flexibility, and prevents melting of duplex DNA: implications for DNA recombination. Cell Rep. *18*, 2845–2853.

Burns, K.H. (2017). Transposable elements in cancer. Nat. Rev. Cancer *17*, 415–424.

Carvalho, C.M., and Lupski, J.R. (2016). Mechanisms underlying structural variant formation in genomic disorders. Nat. Rev. Genet. *17*, 224–238.

Cer, R.Z., Donohue, D.E., Mudunuri, U.S., Temiz, N.A., Loss, M.A., Starner, N.J., Halusa, G.N., Volfovsky, N., Yi, M., Luke, B.T., et al. (2013). Non-B DB v2.0: a database of predicted non-B DNA-forming motifs and its associated tools. Nucleic Acids Res. *41*, D94–D100.

Chaganti, R.S., Schonberg, S., and German, J. (1974). A manyfold increase in sister chromatid exchanges in Bloom's syndrome lymphocytes. Proc. Natl. Acad. Sci. USA *71*, 4508–4512.

Chakraborty, U., and Alani, E. (2016). Understanding how mismatch repair proteins participate in the repair/anti-recombination decision. FEMS Yeast Res. *16*, fow071.

Chakraborty, U., George, C.M., Lyndaker, A.M., and Alani, E. (2016). A delicate balance between repair and replication factors regulates recombination between divergent DNA sequences in Saccharomyces cerevisiae. Genetics *202*, 525–540.

Chen, C.C., Avdievich, E., Zhang, Y., Zhang, Y., Wei, K., Lee, K., Edelmann, W., Jasin, M., and LaRocque, J.R. (2017). EXO1 suppresses double-strand break induced homologous recombination between diverged sequences in mammalian cells. DNA Repair (Amst.) *57*, 98–106.

Claij, N., and te Riele, H. (2004). Msh2 deficiency does not contribute to cisplatin resistance in mouse embryonic stem cells. Oncogene *23*, 260–266.

Croteau, D.L., Popuri, V., Opresko, P.L., and Bohr, V.A. (2014). Human RecQ helicases in DNA repair, recombination, and replication. Annu. Rev. Biochem. *83*, 519–552.

Daley, J.M., Jimenez-Sainz, J., Wang, W., Miller, A.S., Xue, X., Nguyen, K.A., Jensen, R.B., and Sung, P. (2017). Enhancement of BLM-DNA2-mediated long-range DNA end resection by CtIP. Cell Rep. *21*, 324–332.

de Koning, A.P., Gu, W., Castoe, T.A., Batzer, M.A., and Pollock, D.D. (2011). Repetitive elements may comprise over two-thirds of the human genome. PLoS Genet. *7*, e1002384.

Deininger, P. (2011). Alu elements: know the SINEs. Genome Biol. *12*, 236.

Deininger, P.L., and Batzer, M.A. (1999). Alu repeats and human disease. Mol. Genet. Metab. *67*, 183–193.

Deshpande, R.A., Lee, J.H., Arora, S., and Paull, T.T. (2016). Nbs1 converts the human Mre11/Rad50 nuclease complex into an endo/exonuclease machine specific for protein-DNA adducts. Mol. Cell *64*, 593–606.

Duxin, J.P., Moore, H.R., Sidorova, J., Karanja, K., Honaker, Y., Dao, B., Piwnica-Worms, H., Campbell, J.L., Monnat, R.J., Jr., and Stewart, S.A. (2012). Okazaki fragment processing-independent role for human Dna2 enzyme during DNA replication. J. Biol. Chem. *287*, 21980–21991.

Elliott, B., and Jasin, M. (2001). Repair of double-strand breaks by homologous recombination in mismatch repair-defective mammalian cells. Mol. Cell. Biol. *21*, 2671–2682.

Ellis, N.A., Groden, J., Ye, T.Z., Straughen, J., Lennon, D.J., Ciocci, S., Proytcheva, M., and German, J. (1995). The Bloom's syndrome gene product is homologous to RecQ helicases. Cell *83*, 655–666.

Fishel, R., Lescoe, M.K., Rao, M.R., Copeland, N.G., Jenkins, N.A., Garber, J., Kane, M., and Kolodner, R. (1993). The human mutator gene homolog MSH2 and its association with hereditary nonpolyposis colon cancer. Cell *75*, 1027–1038.

Forment, J.V., Walker, R.V., and Jackson, S.P. (2012). A high-throughput, flow cytometry-based method to quantify DNA-end resection in mammalian cells. Cytometry A *81*, 922–928.

Goldfarb, T., and Alani, E. (2005). Distinct roles for the Saccharomyces cerevisiae mismatch repair proteins in heteroduplex rejection, mismatch repair and nonhomologous tail removal. Genetics *169*, 563–574.

Gratia, M., Rodero, M.P., Conrad, C., Bou Samra, E., Maurin, M., Rice, G.I., Duffy, D., Revy, P., Petit, F., Dale, R.C., et al. (2019). Bloom syndrome protein restrains innate immune sensing of micronuclei by cGAS. J. Exp. Med. *216*, 1199–1213.

Gravel, S., Chapman, J.R., Magill, C., and Jackson, S.P. (2008). DNA helicases Sgs1 and BLM promote DNA double-strand break resection. Genes Dev. *22*, 2767–2772.

Grimme, J.M., Honda, M., Wright, R., Okuno, Y., Rothenberg, E., Mazin, A.V., Ha, T., and Spies, M. (2010). Human Rad52 binds and wraps single-stranded DNA and mediates annealing via two hRad52-ssDNA complexes. Nucleic Acids Res. *38*, 2917–2930.

Gunn, A., and Stark, J.M. (2012). I-SceI-based assays to examine distinct repair outcomes of mammalian chromosomal double strand breaks. Methods Mol. Biol. *920*, 379–391.

Haber, J.E. (2018). DNA repair: the search for homology. BioEssays *40*, e1700229.

Hengel, S.R., Spies, M.A., and Spies, M. (2017). Small-molecule inhibitors targeting DNA repair and DNA repair deficiency in research and cancer therapy. Cell Chem. Biol. *24*, 1101–1119.

Hu, Q., Lu, H., Wang, H., Li, S., Truong, L., Li, J., Liu, S., Xiang, R., and Wu, X. (2019). Break-induced replication plays a prominent role in long-range repeat-mediated deletion. EMBO J. *38*, e101751.

Jain, S., Sugawara, N., Lydeard, J., Vaze, M., Tanguy Le Gac, N., and Haber, J.E. (2009). A recombination execution checkpoint regulates the choice of homologous recombination pathway during DNA double-strand break repair. Genes Dev. *23*, 291–303.

Johnson, R.D., and Jasin, M. (2000). Sister chromatid gene conversion is a prominent double-strand break repair pathway in mammalian cells. EMBO J. *19*, 3398–3407.

Karanja, K.K., Cox, S.W., Duxin, J.P., Stewart, S.A., and Campbell, J.L. (2012). DNA2 and EXO1 in replication-coupled, homology-directed repair and in the interplay between HDR and the FA/BRCA network. Cell Cycle *11*, 3983–3996.

Kelso, A.A., Lopezcolorado, F.W., Bhargava, R., and Stark, J.M. (2019). Distinct roles of RAD52 and POLQ in chromosomal break repair and replication stress response. PLoS Genet. *15*, e1008319.

Kolomietz, E., Meyn, M.S., Pandita, A., and Squire, J.A. (2002). The role of Alu repeat clusters as mediators of recurrent chromosomal aberrations in tumors. Genes Chromosomes Cancer *35*, 97–112.

Krenning, L., van den Berg, J., and Medema, R.H. (2019). Life or death after a break: what determines the choice? Mol. Cell *76*, 346–358.

Larocque, J.R., and Jasin, M. (2010). Mechanisms of recombination between diverged sequences in wild-type and BLM-deficient mouse and human cells. Mol. Cell. Biol. *30*, 1887–1897.

LaRocque, J.R., Stark, J.M., Oh, J., Bojilova, E., Yusa, K., Horie, K., Takeda, J., and Jasin, M. (2011). Interhomolog recombination and loss of heterozygosity in wild-type and Bloom syndrome helicase (BLM)-deficient mammalian cells. Proc. Natl. Acad. Sci. USA *108*, 11971–11976.

Li, Z., Liu, B., Jin, W., Wu, X., Zhou, M., Liu, V.Z., Goel, A., Shen, Z., Zheng, L., and Shen, B. (2018). hDNA2 nuclease/helicase promotes centromeric DNA replication and genome stability. EMBO J. *37*, e96729.

Lo, Y.C., Paffett, K.S., Amit, O., Clikeman, J.A., Sterk, R., Brenneman, M.A., and Nickoloff, J.A. (2006). Sgs1 regulates gene conversion tract lengths and crossovers independently of its helicase activity. Mol. Cell. Biol. *26*, 4086–4094.

Lu, R., O'Rourke, J.J., Sobinoff, A.P., Allen, J.A.M., Nelson, C.B., Tomlinson, C.G., Lee, M., Reddel, R.R., Deans, A.J., and Pickett, H.A. (2019). The FANCM-BLM-TOP3A-RMI complex suppresses alternative lengthening of telomeres (ALT). Nat. Commun. *10*, 2252.

Makharashvili, N., and Paull, T.T. (2015). CtIP: A DNA damage response protein at the intersection of DNA metabolism. DNA Repair (Amst.) *32*, 75–81.

Masuda-Sasa, T., Polaczek, P., Peng, X.P., Chen, L., and Campbell, J.L. (2008). Processing of G4 DNA by Dna2 helicase/nuclease and replication protein A (RPA) provides insights into the mechanism of Dna2/RPA substrate recognition. J. Biol. Chem. *283*, 24359–24373.

Mendez-Dorantes, C., Bhargava, R., and Stark, J.M. (2018). Repeat-mediated deletions can be induced by a chromosomal break far from a repeat, but multiple pathways suppress such rearrangements. Genes Dev. *32*, 524–536.

Mimitou, E.P., and Symington, L.S. (2008). Sae2, Exo1 and Sgs1 collaborate in DNA double-strand break processing. Nature *455*, 770–774.

Morales, M.E., White, T.B., Streva, V.A., DeFreece, C.B., Hedges, D.J., and Deininger, P.L. (2015). The contribution of alu elements to mutagenic DNA double-strand break repair. PLoS Genet. *11*, e1005016.

Morales, M.E., Kaul, T., and Deininger, P. (2018). Long-distance relationships: suppression of repeat-mediated deletions. Trends Genet. *34*, 572–574.

Moynahan, M.E., Pierce, A.J., and Jasin, M. (2001). BRCA2 is required for homology-directed repair of chromosomal breaks. Mol. Cell *7*, 263–272.

Muñoz, M.C., Laulier, C., Gunn, A., Cheng, A., Robbiani, D.F., Nussenzweig, A., and Stark, J.M. (2012). RING finger nuclear factor RNF168 is important for defects in homologous recombination caused by loss of the breast cancer susceptibility factor BRCA1. J. Biol. Chem. *287*, 40618–40628.

Muñoz, M.C., Yanez, D.A., and Stark, J.M. (2014). An RNF168 fragment defective for focal accumulation at DNA damage is proficient for inhibition of homologous recombination in BRCA1 deficient cells. Nucleic Acids Res. *42*, 7720–7733.

Myler, L.R., Gallardo, I.F., Zhou, Y., Gong, F., Yang, S.H., Wold, M.S., Miller, K.M., Paull, T.T., and Finkelstein, I.J. (2016). Single-molecule imaging reveals the mechanism of Exo1 regulation by single-stranded DNA binding proteins. Proc. Natl. Acad. Sci. USA *113*, E1170–E1179.

Nik-Zainal, S., and Hall, B.A. (2019). Cellular survival over genomic perfection. Science *366*, 802–803.

Nimonkar, A.V., Genschel, J., Kinoshita, E., Polaczek, P., Campbell, J.L., Wyman, C., Modrich, P., and Kowalczykowski, S.C. (2011). BLM-DNA2-RPA-MRN and EXO1-BLM-RPA-MRN constitute two DNA end resection machineries for human DNA break repair. Genes Dev. *25*, 350–362.

Nitiss, J.L. (2009). Targeting DNA topoisomerase II in cancer chemotherapy. Nat. Rev. Cancer *9*, 338–350.

Orans, J., McSweeney, E.A., Iyer, R.R., Hast, M.A., Hellinga, H.W., Modrich, P., and Beese, L.S. (2011). Structures of human exonuclease 1 DNA complexes suggest a unified mechanism for nuclease family. Cell *145*, 212–223.

Panier, S., Maric, M., Hewitt, G., Mason-Osann, E., Gali, H., Dai, A., Labadorf, A., Guervilly, J.H., Ruis, P., Segura-Bayona, S., et al. (2019). SLX4IP antagonizes promiscuous BLM activity during ALT maintenance. Mol. Cell *76*, 27–43.e11.

Patel, D.S., Misenko, S.M., Her, J., and Bunting, S.F. (2017). BLM helicase regulates DNA repair by counteracting RAD51 loading at DNA double-strand break sites. J. Cell Biol. *216*, 3521–3534.

Pavlicek, A., Noskov, V.N., Kouprina, N., Barrett, J.C., Jurka, J., and Larionov, V. (2004). Evolution of the tumor suppressor BRCA1 locus in primates: implications for cancer predisposition. Hum. Mol. Genet. *13*, 2737–2751.

Pérez-Cabornero, L., Borrás Flores, E., Infante Sanz, M., Velasco Sampedro, E., Acedo Becares, A., Lastra Aras, E., Cuevas González, J., Pineda Riu, M., Ramón y Cajal Asensio, T., Capellá Munar, G., et al. (2011). Characterization of new founder Alu-mediated rearrangements in MSH2 gene associated with a Lynch syndrome phenotype. Cancer Prev. Res. (Phila.) *4*, 1546–1555.

Piazza, A., Shah, S.S., Wright, W.D., Gore, S.K., Koszul, R., and Heyer, W.D. (2019). Dynamic processing of displacement loops during recombinational DNA repair. Mol. Cell *73*, 1255–1266.e4.

Pinto, C., Kasaciunaite, K., Seidel, R., and Cejka, P. (2016). Human DNA2 possesses a cryptic DNA unwinding activity that functionally integrates with BLM or WRN helicases. eLife *5*, e18574.

Ran, F.A., Hsu, P.D., Wright, J., Agarwala, V., Scott, D.A., and Zhang, F. (2013). Genome engineering using the CRISPR-Cas9 system. Nat. Protoc. *8*, 2281–2308.

Rein, K., Yanez, D.A., Terré, B., Palenzuela, L., Aivio, S., Wei, K., Edelmann, W., Stark, J.M., and Stracker, T.H. (2015). EXO1 is critical for embryogenesis and the DNA damage response in mice with a hypomorphic Nbs1 allele. Nucleic Acids Res. *43*, 7371–7387.

Rong, S.B., Väliaho, J., and Vihinen, M. (2000). Structural basis of Bloom syndrome (BS) causing mutations in the BLM helicase domain. Mol. Med. *6*, 155–164.

Russo, M., Crisafulli, G., Sogari, A., Reilly, N.M., Arena, S., Lamba, S., Bartolini, A., Amodio, V., Magrì, A., Novara, L., et al. (2019). Adaptive mutability of colorectal cancers in response to targeted therapies. Science *366*, 1473–1480.

Sartori, A.A., Lukas, C., Coates, J., Mistrik, M., Fu, S., Bartek, J., Baer, R., Lukas, J., and Jackson, S.P. (2007). Human CtIP promotes DNA end resection. Nature *450*, 509–514.

Silva, B., Pentz, R., Figueira, A.M., Arora, R., Lee, Y.W., Hodson, C., Wischnewski, H., Deans, A.J., and Azzalin, C.M. (2019). FANCM limits ALT activity by restricting telomeric replication stress induced by deregulated BLM and R-loops. Nat. Commun. *10*, 2253.

Song, X., Beck, C.R., Du, R., Campbell, I.M., Coban-Akdemir, Z., Gu, S., Breman, A.M., Stankiewicz, P., Ira, G., Shaw, C.A., and Lupski, J.R. (2018). Predicting human genes susceptible to genomic instability associated with *Alu/Alu*-mediated rearrangements. Genome Res. *28*, 1228–1242.

Soniat, M.M., Myler, L.R., Kuo, H.C., Paull, T.T., and Finkelstein, I.J. (2019). RPA phosphorylation inhibits DNA resection. Mol. Cell *75*, 145–153.e5.

Spell, R.M., and Jinks-Robertson, S. (2004). Examination of the roles of Sgs1 and Srs2 helicases in the enforcement of recombination fidelity in Saccharomyces cerevisiae. Genetics *168*, 1855–1865.

Stark, J.M., Pierce, A.J., Oh, J., Pastink, A., and Jasin, M. (2004). Genetic steps of mammalian homologous repair with distinct mutagenic consequences. Mol. Cell. Biol. *24*, 9305–9316.

Sturzenegger, A., Burdova, K., Kanagaraj, R., Levikova, M., Pinto, C., Cejka, P., and Janscak, P. (2014). DNA2 cooperates with the WRN and BLM RecQ helicases to mediate long-range DNA end resection in human cells. J. Biol. Chem. *289*, 27314–27326.

Sugawara, N., Goldfarb, T., Studamire, B., Alani, E., and Haber, J.E. (2004). Heteroduplex rejection during single-strand annealing requires Sgs1 helicase and mismatch repair proteins Msh2 and Msh6 but not Pms1. Proc. Natl. Acad. Sci. USA *101*, 9315–9320.

Symington, L.S., and Gautier, J. (2011). Double-strand break end resection and repair pathway choice. Annu. Rev. Genet. *45*, 247–271.

Thangavel, S., Berti, M., Levikova, M., Pinto, C., Gomathinayagam, S., Vujanovic, M., Zellweger, R., Moore, H., Lee, E.H., Hendrickson, E.A., et al. (2015). DNA2 drives processing and restart of reversed replication forks in human cells. J. Cell Biol. *208*, 545–562.

Tomimatsu, N., Mukherjee, B., Deland, K., Kurimasa, A., Bolderson, E., Khanna, K.K., and Burma, S. (2012). Exo1 plays a major role in DNA end resection in humans and influences double-strand break repair and damage signaling decisions. DNA Repair (Amst.) *11*, 441–448.

Treangen, T.J., and Salzberg, S.L. (2011). Repetitive DNA and next-generation sequencing: computational challenges and solutions. Nat. Rev. Genet. *13*, 36–46.

Tutt, A., Bertwistle, D., Valentine, J., Gabriel, A., Swift, S., Ross, G., Griffin, C., Thacker, J., and Ashworth, A. (2001). Mutation in Brca2 stimulates error-prone homology-directed repair of DNA double-strand breaks occurring between repeated sequences. EMBO J. *20*, 4704–4716.

Waldman, A.S., and Liskay, R.M. (1988). Dependence of intrachromosomal recombination in mammalian cells on uninterrupted homology. Mol. Cell. Biol. *8*, 5350–5357.

Wang, Y., Li, S., Smith, K., Waldman, B.C., and Waldman, A.S. (2016). Intrachromosomal recombination between highly diverged DNA sequences is enabled in human cells deficient in Bloom helicase. DNA Repair (Amst.) *41*, 73–84.

Wang, H., Li, S., Oaks, J., Ren, J., Li, L., and Wu, X. (2018a). The concerted roles of FANCM and Rad52 in the protection of common fragile sites. Nat. Commun. *9*, 2791.

Wang, H., Li, S., Zhang, H., Wang, Y., Hao, S., and Wu, X. (2018b). BLM prevents instability of structure-forming DNA sequences at common fragile sites. PLoS Genet. *14*, e1007816.

Weinstein, J., and Rothstein, R. (2008). The genetic consequences of ablating helicase activity and the Top3 interaction domain of Sgs1. DNA Repair (Amst.) *7*, 558–571.

Yusa, K., Horie, K., Kondoh, G., Kouno, M., Maeda, Y., Kinoshita, T., and Takeda, J. (2004). Genome-wide phenotype analysis in ES cells by regulated disruption of Bloom's syndrome gene. Nature *429*, 896–899.

Zhou, Y., Caron, P., Legube, G., and Paull, T.T. (2014). Quantitation of DNA double-strand break resection intermediates in human cells. Nucleic Acids Res. *42*, e19.

Zhu, Z., Chung, W.H., Shim, E.Y., Lee, S.E., and Ira, G. (2008). Sgs1 helicase and two nucleases Dna2 and Exo1 resect DNA double-strand break ends. Cell *134*, 981–994.

STAR★METHODS

KEY RESOURCES TABLE

REAGENT or RESOURCE	SOURCE	IDENTIFIER
Antibodies		
Rat monoclonal anti-RPA32	Cell Signaling	Cat#2208; RRID:AB_2238543
Rabbit polyclonal anti-BLM	Bethyl Laboratories	Cat#A300-110A; RRID:AB_2064794
Rabbit polyclonal anti-BRCA2	Abcam	Cat#ab27976; RRID:AB_2067760
Mouse monoclonal anti-CtIP	Active Motif	Cat#61141, RRID:AB_2714164
Rabbit polyclonal anti-EXO1	Bethyl Laboratories	Cat#A302-640A; RRID:AB_10567122
Rabbit polyclonal anti-RAD52	ABclonal	Cat#A3077; RRID:AB_276488
Rabbit polyclonal anti-MSH2	Bethyl Laboratories	Cat# A300-452A; RRID:AB_420918
Rabbit polyclonal anti-ACTIN	Sigma-Aldrich	Cat#A2066; RRID:AB_476693
Mouse monoclonal anti-FLAG HRP	Sigma-Aldrich	Cat#A8592; RRID:AB_439702
Goat anti-Rat IgG, Alexa Fluor 488	Thermo Fisher	Cat#A-11006; RRID:AB_141373
Mouse monoclonal Anti-BrdU, FITC	Becton Dickinson	Cat#556028; RRID:AB_396304
Goat polyclonal Anti-Mouse IgG, HRP	Abcam	Cat#ab205719; RRID:AB_2755049
Goat polyclonal Anti-Rabbit IgG, HRP	Abcam	Cat#ab205718
Chemicals, Peptides, and Recombinant Proteins		
Doxycycline hyclate	Sigma Aldrich	Cat#D9891
Lipfectamine 2000	Themo Fisher	Cat#11668019
Lipfectamine RNAiMAX	Thermo Fisher	Cat#13778150
BD Cytofix/Cytoperm buffer	Becton Dickinson	51-2090KZ
BD Perm/Wash buffer	Becton Dickinson	51-2091KZ
RNase A	Sigma Aldrich	Cat#R4642
DAPI (4′,6-Diamidino-2-phenylindole dihydrochloride)	Sigma Aldrich	Cat#D8417
MMLV Reverse Transcriptase	Promega	Cat#M170A
SYBR Select Master Mix	Thermo Fisher	Cat#4472908
Etoposide	Sigma Aldrich	Cat#E1383
propidium iodine	Sigma Aldrich	Cat#P4170
bromodeoxyuridine	Becton Dickinson	Cat#51-2420KC
Mouse Leukemia Inhibitory Factor	Gemini	Cat#400-495 10ˆ7
2-Mercaptoethanol	Sigma Aldrich	Cat#M6250
DMEM high glucose supplemented with L-Glutamine	Corning	Cat#10-017-CV
non-essential amino acids	Irvine Scientific	Cat#9304
Penicillin/Streptomycin	Corning	Cat#30-002-C1
Plasmocin	Invivogen	Cat#ant-mpt
hygromycin	Thermo Fisher	Cat#10687010
puromycin	Sigma Aldrich	Cat#P8833
Protease inhibitor	Roche	Cat#11836153001
Igepal	Sigma Aldrich	Cat#I8896
Dimethyl sulfoxide	Sigma Aldrich	Cat#D2650
Triton X-100	USB	Cat#22686
Gelatin	Sigma Aldrich	Cat#G1890

(*Continued on next page*)

Continued		
REAGENT or RESOURCE	SOURCE	IDENTIFIER
Critical Commercial Assays		
RNAeasy Plus Kit	QIAGEN	Cat#74134
ECL western blotting substrate	Thermo Fisher	Cat#32106
Surveyor Mutation Detection Kit	Integrated DNA Technologies	Cat#706020
Experimental Models: Cell Lines		
WT mESC J1 strain	ATCC	Cat#SCRC-1010
Blm$^{tet/tet}$ mESC line	Yusa et al., 2004	N/A
Rad52−/− mESC line	Mendez-Dorantes et al., 2018	N/A
Msh2−/− mESC line	Claij and te Riele, 2004	N/A
Rad52−/−*Msh2*−/− mESC line	This study	N/A
Exo1−/− mESC line	Chen et al., 2017	N/A
Oligonucleotides		
Oligonucleotide sequences for sgRNAs, siRNA, and PCR primers	See Table S1 for sequences	N/A
Recombinant DNA		
RMD-GFP reporter plasmid	Mendez-Dorantes et al., 2018	N/A
1%-RMD-GFP reporter plasmid	Mendez-Dorantes et al., 2018	N/A
3%-RMD-GFP reporter plasmid	Mendez-Dorantes et al., 2018	N/A
Pim-DR-GFP reporter plasmid	Moynahan et al., 2001	N/A
px330 sgRNA/Cas9 expression vector	Addgene	Cat#42230
pCAGGS-BSKX empty expression vector	Gunn and Stark, 2012	N/A
pgk-puro	Gunn and Stark, 2012	N/A
pCAGGS-NZE-GFP (GFP expression vector)	Gunn and Stark, 2012	N/A
pCAGGS-EXO1 WT	This study	N/A
pCAGGS-EXO1-D173A	This study	N/A
pCAGGS-3xFlag-DNA2 WT	This study	N/A
pCAGGS-3xFlag-DNA2 D277A	This study	N/A
pCAGGS-3xFlag-DNA2 K654A	This study	N/A
pCAGGS-3xFlag-DNA2 D277A/K654A	This study	N/A
pCAGGS-BLM WT	This study	N/A
pCAGGS-BLM K695A/K795A	This study	N/A
dsRED2-N1	Clontech	Cat#632406
Software and Algorithms		
GraphPad Prism	GraphPad	N/A
non-B DNA Motif Search Tool	Cer et al., 2013	N/A

LEAD CONTACT AND MATERIALS AVAILABILITY

Further information and requests for resources and reagents should be directed to and will be fulfilled by the Lead Contact, Jeremy M Stark (jstark@coh.org). All unique/stable reagents (e.g., plasmids and cell lines) generated in this study are available from the Lead Contact with a completed Materials Transfer Agreement, as needed.

EXPERIMENTAL MODEL AND SUBJECT DETAILS

The model cell line for this study is male mESCs, cultured at 37°C and 5% CO_2 on tissue culture dishes coated with 0.1% Gelatin. Standard media for mESCs was used: 500 mL DMEM high glucose supplemented with L-Glutamine, 6 mL 100X non-essential amino acids, 75 mL Stasis Fetal Bovine Serum, 45 μL of Mouse Leukemia Inhibitory Factor, 4.3 μL 2-Mercaptoethanol, 6 mL 100X Penicillin/Streptomycin, and 120 μL Plasmocin.

Several mESC lines were previously described: WT J1 (Mendez-Dorantes et al., 2018), *Blm*$^{tet/tet}$ (Yusa et al., 2004), *Msh2*−/− (Claij and te Riele, 2004; Mendez-Dorantes et al., 2018), *Exo1*−/− (Chen et al., 2017), and *Rad52*−/− (Mendez-Dorantes et al., 2018). The *Rad52*−/−*Msh2*−/− mESC line was derived from the *Rad52*−/− cell line, using two Cas9-mediated DSBs to induce a deletion in *Msh2* using two sgRNAs (Msh2 sgRNA 1 and 2, Table S1). Cells were transfected with these plasmids and dsRED2-N1, as with the reporter assays (see below), and sorted for dsRED+ cells as described (Kelso et al., 2019), which were plated at low density. Clones were first screened for a deletion mutation with PCR (primers Msh2 screening P1 and P2, Table S1), and then by MSH2 immunoblotting.

METHOD DETAILS

Reporter integration and recombinant DNA

Cas9 and sgRNAs were expressed from the px330 plasmid (Addgene 42230, deposited by Dr. Feng Zhang) (Ran et al., 2013). The reporter plasmids RMD-GFP, 1%-RMD-GFP, 3%-RMD-GFP, and Pim-DR-GFP were previously described (Mendez-Dorantes et al., 2018; Moynahan et al., 2001). The reporter plasmids were integrated into the *Pim1* locus of mESCs using electroporation of linerarized plasmid, selection in hygromycin, and screening by PCR, as described previously (Bennardo et al., 2008). For the *Blm*$^{tet/tet}$ cells, we included in the electroporation a px330 plasmid with an sgRNA targeting the *Pim1* locus (Pim1 sgRNA 1, Table S1) to promote gene targeting. For these *Blm*$^{tet/tet}$ reporter cell lines, the untargeted *Pim1* locus surrounding the sgRNA target site was amplified for sequencing with primers Pim1 screening P1 and P2 (Table S1).

The sgRNA target sequences for inducing DSBs in the reporters were described previously (Mendez-Dorantes et al., 2018; Muñoz et al., 2014) (Table S1). The plasmids pCAGGS-V5-RAD52, pCAGGS-MSH2, pCAGGS-NZE-GFP (GFP expression vector), pgk-puro, and pCAGGS-BSKX empty vector (EV) were described previously (Gunn and Stark, 2012; Mendez-Dorantes et al., 2018). pCAGGS-EXO1 was generated by inserting the EXO1 coding region from Mammalian Gene Collection clone number 5686 into pCAGGS-BSKX. pCAGGS-3xFlag-DNA2 WT, D277A, K654A, and D277A/K654A were generated from Addgene plasmids # 31955, 31956, 31957, and 31958, respectively, which were deposited by Dr. Sheila Stewart (Duxin et al., 2012; Li et al., 2018; Pinto et al., 2016). pCAGGS-BLM WT was generated from Addgene plasmid #127641, which was deposited by Dr. Nicolas Manel (Gratia et al., 2019). The EXO1-D173A (Orans et al., 2011) and BLM-K695A/795A (Wang et al., 2018b) mutants were generated using the QuikChange kit. For siRNA reagents, we used the following reagents from Dharmacon (Table S1): non-targeting siCTRL (D-001810-01-20), siBLM (pool of 4, D-061987-01, −02, −03, −04), siDNA2 (pool of 4, D-062864-01, −02, −03, −04), siCtIP (pool of 4, D-055713-14, −15, −16, −17) and siBRCA2 (pool of 4, J-042993-05, −06, −07, −08). The pools of siRNAs contain each of the four in equal amounts.

RMD and HDR reporter assays

For the RMD assays including siRNA, mESC cells were seeded on a mixture of 3.75 pmol of each siRNA pool using RNAiMAX at a cell density of 0.5 × 10^5 cells per well of a 24-well plate, with 0.5 ml of antibiotic-free media. The next day, each well was transfected with 200 ng of each sgRNA/Cas9 plasmid plus 3.75 pmol of each siRNA pool using Lipofectamine 2000, with 0.5 ml of antibiotic-free media. For the RMD assays with complementation vectors, transfections with siRNA included 200 ng of complementation vector, and transfections without siRNA included 100 ng of complementation vector. The EV control (pCAGGS-BSKX) was used to ensure equal total plasmid concentration per experiment. For RMD assays with *Blm*$^{tet/tet}$ mESCs, cells were seeded at 0.25 × 10^5 cells on 24-well plates in either DMSO or Dox (1 μg/mL) two days prior to transfection with the sgRNA/Cas9 plasmids and 200 ng of EV, followed by DMSO or Dox (1 μg/mL) treatment until analysis. For RNAi experiments with the *Blm*$^{tet/tet}$ mESCs, 5 pmol of siRNA was used in place of EV. For DR-GFP analysis with siBRCA2, cells were transfected with a px330 plasmid expressing an sgRNA targeting DR-GFP (Table S1) (Muñoz et al., 2012), 200 ng of EV, and 5 pmol of siRNA. For the DR-GFP experiment in *Blm*$^{tet/tet}$ mESCs, cells were plated and treated as for the RMD assays. Each experiment included parallel transfections with the GFP expression vector (pCAGGS-NZE-GFP) to normalize repair frequencies to transfection efficiency. For all reporter assays, three days after transfection, cells were analyzed by flow cytometry using a CyAn-ADP (Dako), as described (Gunn and Stark, 2012).

End resection assay

Cells were pre-treated with siRNAs using RNAiMAX transfection, using the same concentration of siRNA as for the reporter assays, but scaled four-fold to a well of a 6-well plate, and cultured for two days prior to a one-hour treatment with 3 μM Etoposide or vehicle control (DMSO). Cells were then assayed for chromatin-bound (i.e., mild-detergent resistant) RPA staining, as described (Forment et al., 2012). Specifically, cells were detergent extracted in 100 μL of 0.2% Triton X-100 in PBS on ice for 10 minutes, and washed with 1 mL of BSA-PBS (0.1% BSA in PBS). Next, cells were fixed with 100 μL BD Cytofix/Cytoperm buffer for 15 min, washed with BSA-PBS, and incubated in 50 μL of BD Perm/Wash buffer with 1:800 rat monoclonal anti-RPA32 antibody for 1 hour. Cells were washed with BSA-PBS and re-suspended in 50 μL BD Perm/Wash buffer with 1:200 secondary antibody (goat anti-Rat IgG, Alexa Fluor 488) for 30 min. Finally, cells were washed with BSA-PBS and re-suspended in 0.3 mL PBS with 0.02% sodium azide, 250 μg/ml RNase A, and 2 μg/ml DAPI for 30 min at 37°C. Stained cells were analyzed on a CyAn-ADP (Dako) flow cytometer. For consistent analysis, the gate for high RPA staining was set as the top ~5% RPA+ population of the control (DMSO treated) sample for each cell line and experiment.

Immunoblotting and RT-PCR

For immunoblotting analysis, cells were transfected using the same total siRNA and plasmid concentrations as for the reporter assays, but using EV instead of sgRNA/Cas9 plasmids. For analysis of siBLM treated cells, following the pre-treatment with siRNA using RNAiMAX, cells were transfected with pgk-puro plasmid (1200 ng), EV (400 ng), and siRNA (15 pmol). The next day, cells were re-plated into puromycin (1.5 μg/mL) and cultured for one day to enrich for transfected cells. For *Blm*$^{tet/tet}$ mESCs, cells were pre-treated with DMSO or Dox (1 μg/ mL) for two days until protein extraction. To examine siBRCA2 treated cells, WT or *Blm*$^{tet/tet}$ mESCs, the latter of which were pre-treated with DMSO or Dox as for the reporter assays, were transfected with pgk-puro plasmid (1200 ng), EV (400 ng), and 20 pmol of siRNA. The next day, cells were re-plated into puromycin (3 μg/mL) and cultured for one day to enrich for transfected cells. For BLM, RAD52, CtIP, and MSH2 analysis, cells were lysed using NETN buffer (20 mM Tris at pH 8.0, 100 mM NaCl, 1 mM EDTA, 0.5% Igepal, 1.25 mM DTT, Roche protease inhibitor) with several freeze/thaw cycles. For EXO1, BRCA2, and 3xFlag-DNA2 analysis, cells were lysed in ELB buffer (250 mM NaCl, 5 mM EDTA, 50 mM HEPES, 0.1% Ipegal, Roche protease inhibitor) with sonication (Qsonica, Q800R). Extracts were probed with antibodies for rabbit polyclonal anti-BLM, rabbit polyclonal anti-BRCA2, mouse monoclonal anti-CtIP, rabbit polyclonal anti-EXO1, mouse monoclonal anti-FLAG HRP, rabbit polyclonal anti-RAD52, rabbit polyclonal anti-MSH2, rabbit polyclonal anti-ACTIN, and secondary antibodies (goat polyclonal Anti-Mouse IgG HRP, and goat polyclonal Anti-Rabbit IgG HRP). ECL western blotting substrate was used to develop HRP signals. For quantitative RT-PCR analysis to examine DNA2 mRNA, two days after siRNA treatment, total RNA was isolated with the RNAeasy kit and reverse transcribed with MMLV-RT. The RT reactions were amplified with primers for DNA2 (DNA2 RT-PCR P1 and P2), and ACTIN (ACTIN RT-PCR P1 and P2) (Table S1), using SYBR Select Master Mix and quantified on Applied Biosystems 7900HT. Relative levels of DNA2 mRNA were determined using the cycle threshold (Ct) value for DNA2 for individual PCR reactions subtracted by Ct value for the ACTIN control (ΔCt value), which was then subtracted from the corresponding ΔCt from siCTRL treated cells (ΔΔCt), which was used to calculate $2^{\Delta\Delta Ct}$ for the fold-change.

Cell cycle analysis, Surveyor nuclease assay, and G-quandruplex predictions

To examine cell cycle phase, cells were incubated with 10 μM bromodeoxyuridine (BrdU) for 30 minutes, fixed with 70% Ethanol, and stained with mouse monoclonal anti-BrdU FITC antibody, propidium iodine, and RNase A. Staining was evaluated using a CyAn ADP Analyzer (Dako). Mutagenic end joining frequencies were determined for cells transfected as for the reporter experiments, using the Cas9/sgRNA plasmid targeting the 19 kbp DSB/repeat distance. Genomic DNA was isolated and examined with the Surveyor Mutation Detection Kit, as described, using the primers Surveyor P1 and P2 (Table S1) (Mendez-Dorantes et al., 2018). Predictions for G-quadruplex forming repeats was performed with the non-B DNA Motif Search Tool (Cer et al., 2013).

QUANTIFICATION AND STATISTICAL ANALYSIS

Data are shown as the mean ± standard deviation (SD). Statistical comparisons were performed with unpaired two-tailed Student's t tests with the Holm-Sidak correction for multiple comparisons, without assuming a consistent SD, using GraphPad Prism software. The number of replicates (n), and definition of significance (minimum *P value* $\leq$ 0.05), are each described in the figure legends.

DATA AND CODE AVAILABILITY

The published article includes all datasets generated or analyzed during this study.

Cell Reports, Volume *30*

Supplemental Information

BLM has Contrary Effects on Repeat-Mediated Deletions, based on the Distance of DNA DSBs to a Repeat and Repeat Divergence

Carlos Mendez-Dorantes, L. Jillianne Tsai, Eva Jahanshir, Felicia Wednesday Lopezcolorado, and Jeremy M. Stark

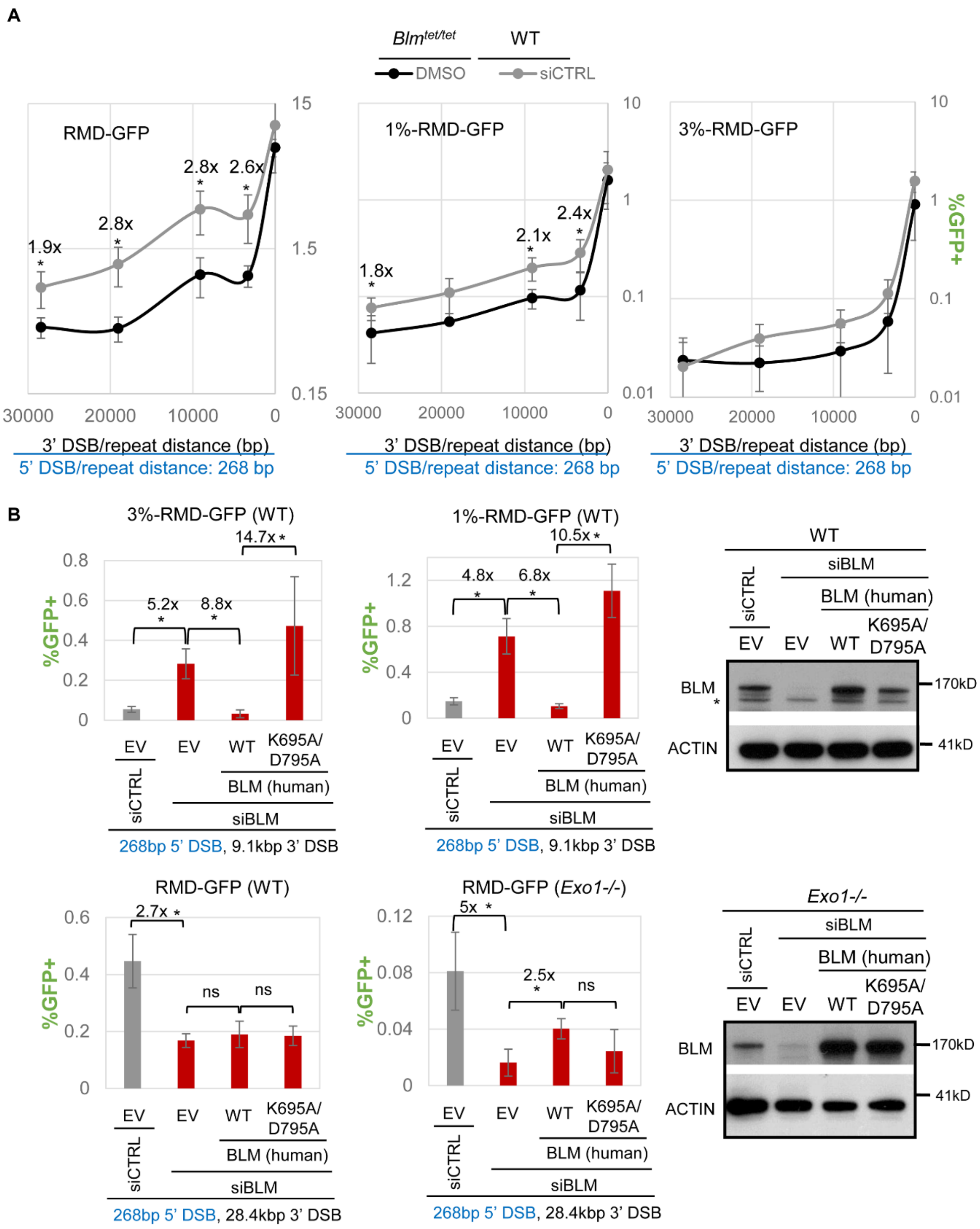

Figure S1. RMD frequencies are reduced in WT vs. *Blm*$^{tet/tet}$mESCs; WT, but not K695A/D795A, human BLM suppresses RMDs between 1% or 3% divergent repeats at the 9.1 kbp 3'-DSB/repeat distance, Related to Figure 1. (A) Comparisons of WT (siCTRL treated) vs. *Blm*$^{tet/tet}$ (DMSO treated) mESCs for each RMD reporter (identical, 1%, and 3%). Frequencies are the same as in Figure 1. N=6. *$P \leq 0.0266$ for WT siCTRL treated vs. *Blm*$^{tet/tet}$ DMSO treated, except at 28.4 kbp between 1% divergent repeats where $P = 0.0487$. (B) Frequencies of specific RMDs for cells treated with siCTRL or siBLM and transfected with various expression vectors (human BLM WT, the K695A/D795A mutant, and EV). WT N=6. *Exo1-/-* N=5. *$P \leq 0.0041$. Immunoblotting analysis of BLM and ACTIN of cells treated with siRNA, and transfected with these vectors. *nonspecific band.

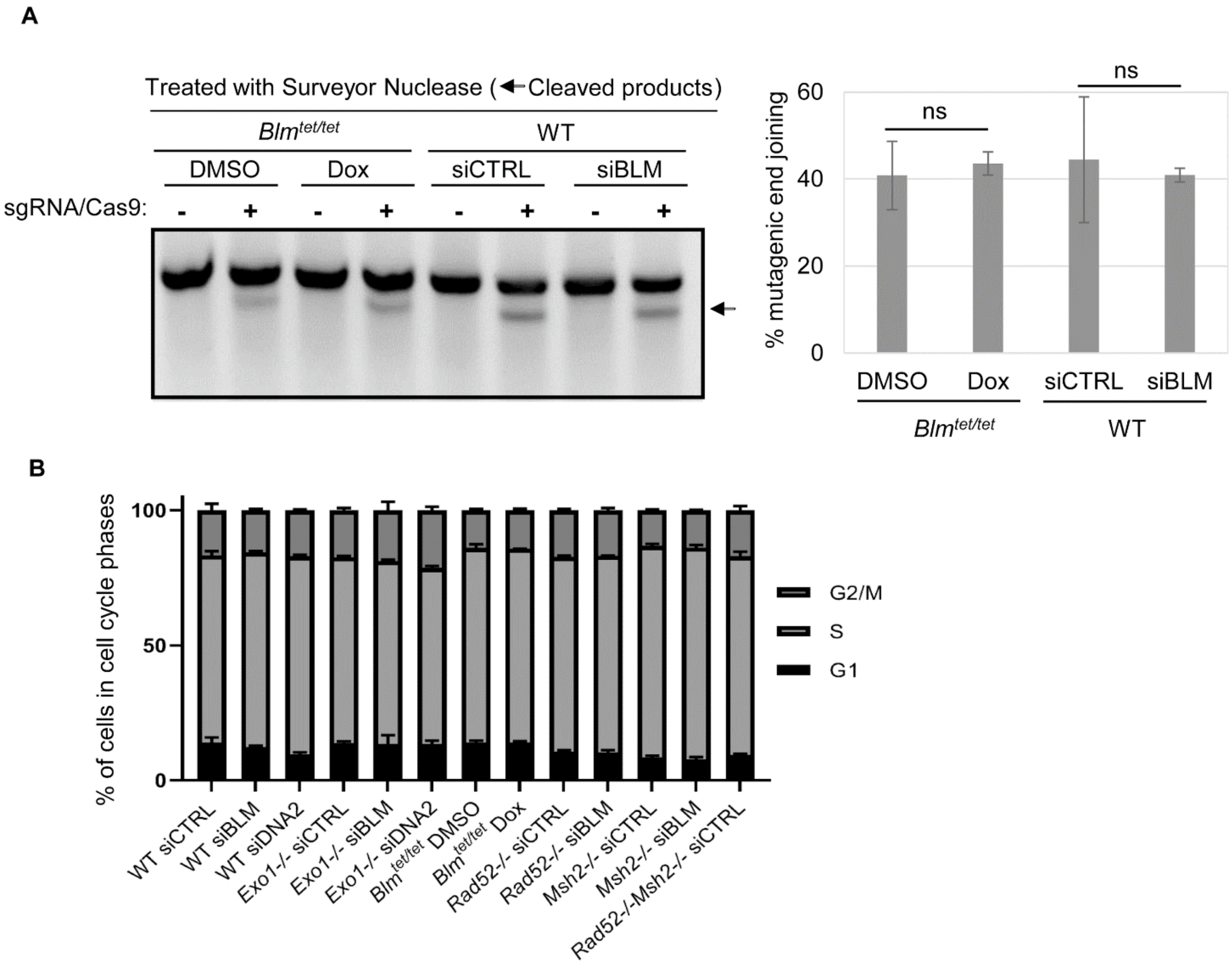

Figure S2. BLM depletion does not obviously affect Cas9-mediated mutagenic end joining; cell cycle profiles are not obviously affected by the genetic depletions/disruptions of various factors, Related to Figure 1. (A) Representative Surveyor nuclease assays from cells transfected with the 19 kbp sgRNA/Cas9 expression plasmid (sgRNA/Cas9). Arrow indicates the Surveyor cleaved product. Shown is the frequency of mutagenic end joining from replicates of this assay, normalized to transfection efficiency. N=3. ns= not significant. (B) Cell cycle profiles (G1, S and G2/M) of the mESC lines under various genetic depletions/disruptions, using BrdU labeling and propidium iodide staining. N=3.

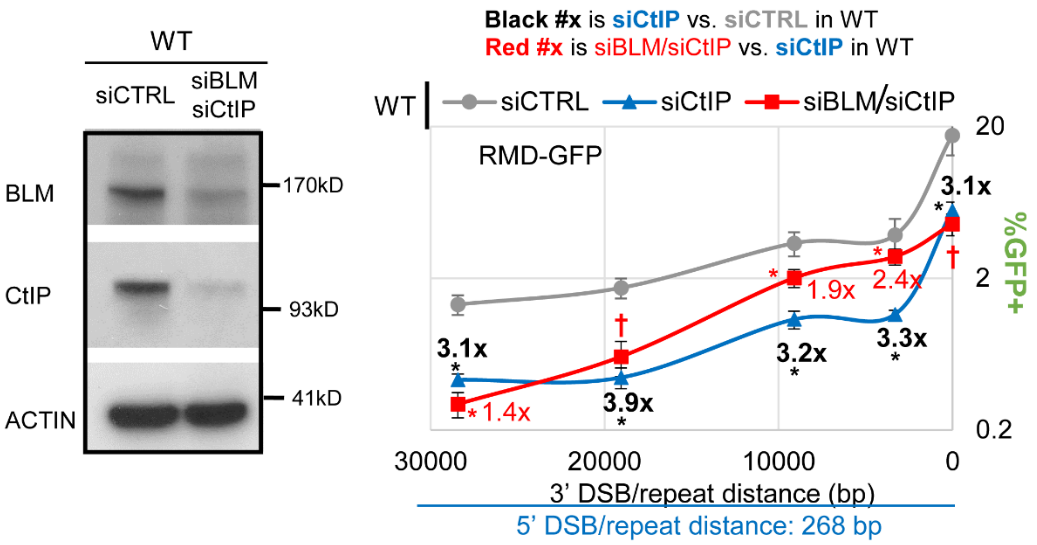

Figure S3. Influence of CtIP depletion alone, and in combination with BLM depletion, on RMDs with identical repeats, Related to Figure 2. RMD frequencies for cells treated with four siRNAs targeting CtIP (siCtIP) alone, or in combination with siBLM (siBLM/siCtIP), along with non-targeting siCTRL. N=6. *$P \leq 0.00275$, or †P-unadjusted≤ 0.041 for the indicated comparisons. Immunoblotting analysis of CtIP, BLM, and ACTIN from cells treated with these siRNAs.

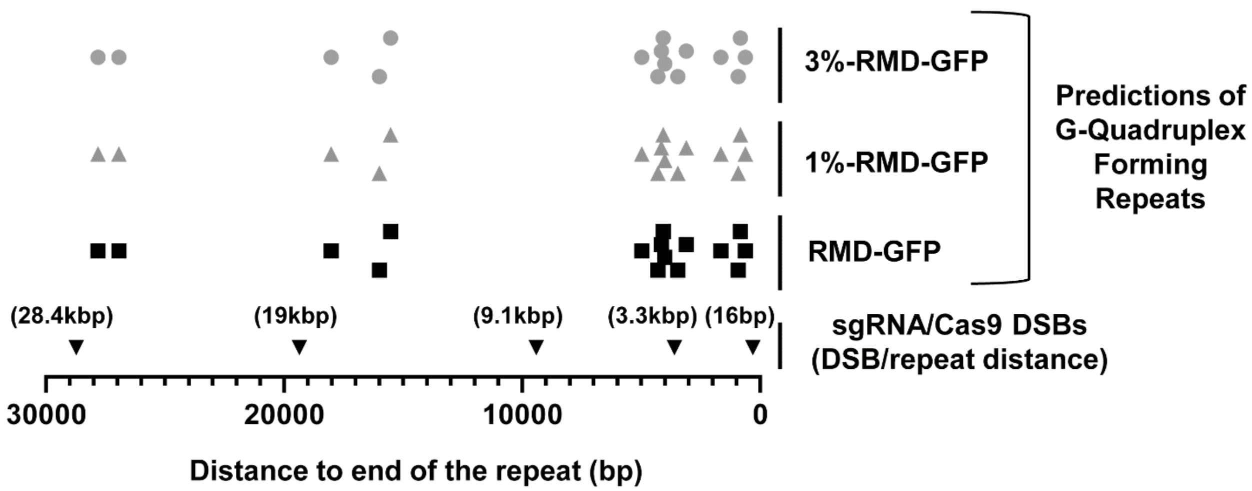

Figure S4. Predictions of G-quadruplex forming repeats in the RMD-GFP reporter assay sequences, Related to Figure 6. Shown are predictions of G-quadruplex forming repeats in the edge of the 28.4 kbp DSB to the end of each repeat sequence (identical, 1% or 3%) in the RMD-GFP reporters, based on the sequence of chromosome 17 from Genome Reference Consortium Mouse Build 38 (GCA_000001635.2), and using the non-B DNA Motif Search Tool (Cer et al., 2013). Also shown are the locations of the sgRNA/Cas9-mediated DSBs.

Table S1. List of oligonucleotide sequences used in this study, Related to STAR Methods.

Reagent	Sequence
DRGFP sgRNA target	5' GGATAACAGGGTAATACCTA
5’268 sgRNA target	5’ GCCATAGGCGTGGGACCTCGT
16 bp sgRNA target	5’ GATCAGAAGCTTAGGGATAAC
3.3 kbp sgRNA target	5’GCGCCGCACCGACACCCTGG
9.1 kbp sgRNA target	5’ GATGTTTGGATTGTATGGCCA
19 kbp sgRNA target	5’ GTCCTGCCATAGCCTTGATG
28.4 kbp sgRNA target	5’ GCGAAGCTAACGACACTAACG
Msh2 sgRNA 1	5' GTTGGAAGGCGCGGCCGAGG
Msh2 sgRNA 2	5’ GACGGTGCGCCTCTTCGACCG
Msh2 screening P1	5’ CGGTGCAGCCTAAGGAGAC
Msh2 screening P2	5’ CGGCCCCATGTACTTGAT
Pim1 sgRNA 1	5’ GCTCGGGCGTCATTAGACTTC
Pim1 screening P1	5’ CTAACCCTCGCCCTCACTCT
Pim1 screening P2	5’ CTGGCTCACCATCAAAGTCC
DNA2 RT-PCR P1	5’ GCGTTCTGCTGACCAGCTAC
DNA2 RT-PCR P2	5’ CTGGATGTCTGGATGGACCT
ACTIN RT-PCR P1	5’ GGCTGTATTCCCCTCCATCG
ACTIN RT-PCR P2	5’ CCAGTTGGTAACAATGCCATGT
Surveyor P1	5’CAGCGAACAGGTCGGTTAAT
Surveyor P2	5’ATCACTTGCTCAAGCACACG
non-targeting siCTRL D-001810-01	5' UGGUUUACAUGUCGACUAA
siBLM D-061987-01 target	5' GACACAAUCUGAAGUACUA
siBLM D-061987-02 target	5 'CUAAAUCUAUGGAGGGUUA
siBLM D-061987-03 target	5' CCUAUGAUAUCGAUAACUU
siBLM D-061987-04 target	5' ACACCUGCGUUAAGUGAUA
siDNA2 D-062864-01 target	5' CGACAUAAGUACUCCACUA
siDNA2 D-062864-02 target	5' CGAGGUCCAUUGCGUCUUU
siDNA2 D-062864-03 target	5' GCACUCCGCCGUUGACAAU
siDNA2 D-062864-04 target	5' AAUAGACGAUGACUUUGGA
siCtIP D-055713-14 target	5' CCUAGACACUGGCGUGAAA
siCtIP D-055713-15 target	5' GCAUUAACCGGCUACGAAA
siCtIP D-055713-16 target	5' AUAUUGAGGUAGUUCGGAA
siCtIP D-055713-17 target	5' AGAUAUGUUUGAUCGGACA
siBRCA2 J-042993-05 target	5' GAACAUAGUUUGGUCAUUA
siBRCA2 J-042993-06 target	5' CUAAGCAGCUCUAUAUAUA
siBRCA2 J-042993-07 target	5' GGAGGUAAAUGAAAUAUGU
siBRCA2 J-042993-08 target	5' GAUCCAGUCUUGAACCAUA
